# Neurobiologically Based Stratification of Recent-Onset Depression and Psychosis: Identification of Two Distinct Transdiagnostic Phenotypes

**DOI:** 10.1016/j.biopsych.2022.03.021

**Published:** 2022-04-12

**Authors:** Paris Alexandros Lalousis, Lianne Schmaal, Stephen J. Wood, Renate L.E.P. Reniers, Nicholas M. Barnes, Katharine Chisholm, Sian Lowri Griffiths, Alexandra Stainton, Junhao Wen, Gyujoon Hwang, Christos Davatzikos, Julian Wenzel, Lana Kambeitz-Ilankovic, Christina Andreou, Carolina Bonivento, Udo Dannlowski, Adele Ferro, Theresa Lichtenstein, Anita Riecher-Rössler, Georg Romer, Marlene Rosen, Alessandro Bertolino, Stefan Borgwardt, Paolo Brambilla, Joseph Kambeitz, Rebekka Lencer, Christos Pantelis, Stephan Ruhrmann, Raimo K.R. Salokangas, Frauke Schultze-Lutter, André Schmidt, Eva Meisenzahl, Nikolaos Koutsouleris, Dominic Dwyer, Rachel Upthegrove, PRONIA Consortium

**Affiliations:** Institute for Mental Health (PAL, SJW, RLEPR, KC, SLG, RU), Centre for Human Brain Health (PAL, RLEPR, SLG, RU), and Institute of Clinical Sciences (RLEPR, NMB), University of Birmingham; Department of Psychology (KC), Aston University; Birmingham Early Interventions Service (RU), Birmingham Women’s and Children’s NHS Foundation Trust, Birmingham, United Kingdom; Orygen (LS, SJW, ASt), Parkville; Centre for Youth Mental Health (LS, SJW, ASt) and Melbourne Neuropsychiatry Centre (CP), University of Melbourne, Melbourne, Victoria, Australia; Perelman School of Medicine (JWen, GH, CD), University of Pennsylvania, Philadelphia, Pennsylvania; Department of Psychiatry and Psychotherapy (JWenz, LK-I, TL, MR, SB), Faculty of Medicine and University Hospital, University of Cologne, Cologne; Institute for Translational Psychiatry (UD, RL), University of Münster, Münster; Department of Psychiatry and Psychotherapy (SB), University of Lübeck, Lübeck; Department of Psychiatry and Psychotherapy (JK, NK, DD), Ludwig Maxmilians University, Munich; Department of Psychiatry and Psychotherapy (FD-L, EM), University of Düsseldorf, Düsseldorf, Germany; Department of Psychiatry (CA, AR-R, PB, RL, SR, ASc), University of Basel, Basel; University Hospital of Child and Adolescent Psychiatry and Psychotherapy (FD-L), University of Bern, Bern, Switzerland; Department of Pathophysiology and Transplantation (CB, AF, GR, PB) and Department of Neurosciences and Mental Health (CB, AF), Fondazione IRCCS Ca’ Granda Ospedale Maggiore Policlinico, University of Milan, Milan; Department of Basic Medical Sciences, Neuroscience and Sense Organs (AB), University of Bari Aldo Moro, Bari, Italy; Department of Psychology and Mental Health (FD-L), Faculty of Psychology, Airlangga University, Surabaya, Indonesia; and the Department of Psychiatry (RKRS), University of Turku, Turku, Finland.

## Abstract

**BACKGROUND:**

Identifying neurobiologically based transdiagnostic categories of depression and psychosis may elucidate heterogeneity and provide better candidates for predictive modeling. We aimed to identify clusters across patients with recent-onset depression (ROD) and recent-onset psychosis (ROP) based on structural neuroimaging data. We hypothesized that these transdiagnostic clusters would identify patients with poor outcome and allow more accurate prediction of symptomatic remission than traditional diagnostic structures.

**METHODS:**

HYDRA (Heterogeneity through Discriminant Analysis) was trained on whole-brain volumetric measures from 577 participants from the discovery sample of the multisite PRONIA study to identify neurobiologically driven clusters, which were then externally validated in the PRONIA replication sample (*n* = 404) and three datasets of chronic samples (Centre for Biomedical Research Excellence, *n* = 146; Mind Clinical Imaging Consortium, *n* = 202; Munich, *n* = 470).

**RESULTS:**

The optimal clustering solution was two transdiagnostic clusters (cluster 1: *n* = 153, 67 ROP, 86 ROD; cluster 2: *n* = 149, 88 ROP, 61 ROD; adjusted Rand index = 0.618). The two clusters contained both patients with ROP and patients with ROD. One cluster had widespread gray matter volume deficits and more positive, negative, and functional deficits (impaired cluster), and one cluster revealed a more preserved neuroanatomical signature and more core depressive symptomatology (preserved cluster). The clustering solution was internally and externally validated and assessed for clinical utility in predicting 9-month symptomatic remission, outperforming traditional diagnostic structures.

**CONCLUSIONS:**

We identified two transdiagnostic neuroanatomically informed clusters that are clinically and biologically distinct, challenging current diagnostic boundaries in recent-onset mental health disorders. These results may aid understanding of the etiology of poor outcome patients transdiagnostically and improve development of stratified treatments.

The current classification of mental disorders is based on a phenomenological approach that uses signs and symptoms to assign a diagnosis. While some diagnoses have high reliability, their usefulness and etiopathogenetic basis is questionable (1–3). For example, there is considerable commonality of symptoms and neurobiological domains across mental disorders, and comorbidity is frequent, with a prevalence of depression in over 40% of people with schizophrenia (4,5) and psychotic symptoms occurring in around 20% of people with depression (6,7).

In terms of brain structure, gray matter volume (GMV) reduction is found in both depression and psychosis, across similar areas such as the anterior insula and the dorsal anterior cingulate cortex (8). This GMV loss has been shown to predate medication exposure, poor functional outcome, neurocognitive deficits, and, in the case of clinical high risk for psychosis, transition to frank illness (5,9–11). Symptoms common to depression and schizophrenia, such as social withdrawal, blunted affect, and alogia, are associated with GMV reduction in the cerebellum, while anhedonia and avolition are negatively correlated with white matter volume of the left anterior limb of the internal capsule and are positively correlated with white matter volume of the left superior longitudinal fasciculus (12).

GMV loss in psychosis and depression may be related to immune dysfunction. Elevated proinflammatory cytokines, potentially resulting from genomic predisposition or response to environmental factors, may lead to activation of astrocytic dysfunction and/or microglia activation, resulting in dendritic pruning and synaptic changes (13–15). Indeed, immune dysfunction is implicated in the etiology of both schizophrenia and depression with cytokines such as interleukin (IL) 6 and C-reactive protein (CRP) detected at elevated levels (16–20), and causality suggested in Mendelian randomization studies of both disorders (17,21).

Currently, diagnoses are not based on underlying brain structure or distinct biological etiology. Patients whose symptoms are potentially caused by different biological processes may be given the same diagnosis and patients whose symptoms are potentially caused by the same biological processes may be provided with a different diagnosis, a practice that may have detrimental effects on outcome prediction development (22–24). Recent research has highlighted this mismatch between diagnostic labels and the clinical and neuroanatomical picture in depression and psychosis (25), and heterogeneity may be particularly pronounced in early stages of developing mental health disorders (26–30). The lack of biological validity of diagnostic groups is thought to be one of the major reasons for poor biomedical translation in psychiatry (31–33).

Only 20% of people with psychosis and 25% of people with depression achieve full remission and response to pharmacological treatment, with the remainder achieving partial response or response without remission (34–37). Biologically driven illness models, able to relate to those at highest risk of poor outcome and chronicity, may allow new and targeted treatments to be delivered early (22). However, recognizing patients on a path to chronic disability, at an early stage, is still difficult in both psychosis and depression (38,39). Previous transdiagnostic research has stressed the need for the use of machine learning (40) and has identified specific patterns of neurocircuit disruption across major psychiatric disorders in emotional reactivity and regulation (41). Reininghaus *et al*., building on previous calls for a dimensional approach to psychosis (42), have shown the use of multidimensional item response modeling to predict psychosis biotypes transcending traditional diagnostic boundaries, with suggestion of an underlying transdiagnostic dimension across psychotic diagnoses (43–45). Recent semi-supervised machine learning studies using neuroanatomical data have identified the presence of an impaired neuroanatomical cluster that is characterized by overall poorer outcomes and functioning in schizophrenia (46) and in youth with internalizing symptoms (47). However, there has not yet been a transdiagnostic investigation of neuroanatomy specifically in depression and psychosis.

Herein, we aimed to identify replicable neuroanatomical clusters across patients with recent-onset depression (ROD) and recent-onset psychosis (ROP). We hypothesized that neuroanatomically derived clusters would be transdiagnostic and related to distinct phenotypes drawn from symptom, neurocognitive, and inflammatory data across both disorders. We further aimed to explore the predictive validity of neuroanatomically identified clusters and externally validated our neuroanatomically based clusters in chronic depression and chronic schizophrenia in an accelerated longitudinal design. We also developed supervised machine learning models to predict symptom remission in ROP and ROD and our neuroanatomically based transdiagnostic clusters. We hypothesized that models developed in neuroanatomically based transdiagnostic clusters will show greater predictive accuracy than those in traditional diagnostic groups.

## METHODS AND MATERIALS

### Study Design

This study uses data from the PRONIA study, an EU-FP7–funded seven-center study, and three external validation datasets. Details of the PRONIA study sites, recruitment protocol, and quality control procedures can be found in Supplement sections 1.1, 1.2 and 1.3 (Tables S1–S3) and a prior publication (48). Data used in this analysis included structural magnetic resonance imaging (MRI), demographic, clinical, neurocognitive, and blood-based biomarker measures. See the Supplement for full details.

### Inclusion and Exclusion Criteria

In brief, participants with ROP had to meet the following criteria: 1) DSM-IV-TR (49) affective or nonaffective psychotic episode (lifetime), 2) criteria for DSM-IV-TR affective or nonaffective psychotic episode fulfilled within the past 3 months, and 3) onset of psychosis within the past 24 months. Patients with ROD had to meet the following criteria: 1) DSM-IV-TR major depressive episode (lifetime), 2) major depressive disorder criteria fulfilled within the past 3 months, and 3) duration of first depressive episode no longer than 24 months. General inclusion criteria can be found in Supplement section 1.5.

### MRI Data Acquisition, Quality Control, and Preprocessing

Participants underwent a multimodal MRI protocol. A minimal harmonization protocol, with which the MR sequences across the different scanners had to comply, and imaging preprocessing is described in Supplement sections 1.3 and 1.4.

### Semi-supervised Machine Learning Analysis

HYDRA (Heterogeneity through Discriminant Analysis) (50) is a semi-supervised machine learning clustering algorithm able to dissect disease heterogeneity by portioning patients based on patterns or transformations between the subpopulations (i.e., clusters) from the patient group and the reference group (i.e., healthy control [HC] subjects) through the use of a convex polytope formed by the combination of multiple linear max-margin classifiers (i.e., support-vector machines [SVMs]) and is able to regress out nuisance covariates, such as age and sex. We used the python version of HYDRA (50) to simultaneously classify patients (ROP + ROD) from HC subjects and partition patients into clusters based on disease-related heterogeneity using structural MRI.

### ComBat Harmonization

To mitigate site effects, prior to applying HYDRA, the R version of the ComBat harmonization technique was used (https://github.com/Jfortin1/ComBatHarmonization). ComBat uses an empirical Bayesian framework that removes variance attributed to scanner differences while retaining disease effects. To further ensure that disease variance would be retained distinct from scanner variance, ComBat was trained on HC subjects and then derived estimates were applied to the patients.

### Model Training

We used whole-volume (GMV and cerebrospinal fluid) brain measures derived from 280 regions of the neuromorphometrics atlas parcellation (CAT12) (four regions excluded due to zero variance) from 577 participants with ROP and ROD and HC subjects (discovery sample of the PRONIA study). Patients with ROP and patients with ROD were grouped together into one patient group. HYDRA was trained using a repeated hold out cross-validation strategy (i.e., 1000 repetitions with 80% of the data for training in each repetition). Age, sex, and total intracranial volume were controlled as covariates. HYDRA was run for 2 to 8 clustering solutions, and adjusted Rand index was used to measure cluster stability. The most stable cluster solution was selected for further analysis. The statistical significance of clusters was assessed in three ways including testing our clustering solution against a Gaussian distribution, which assumes a dimensional severity explanation of our data. Details can be found in Supplement section 1.11.

### Phenotype Characterization

Identified clusters were compared with each other and with HC subjects in terms of neurocognitive performance, blood-based biomarker (IL-1 receptor antagonist, S100B, IL-6, tumor necrosis factor α, CRP, transforming growth factor β, and BDNF [brain-derived neurotrophic factor]) (Supplement section 1.6) and symptom differences (Positive and Negative Syndrome Scale, Beck Depression Inventory, Scale for the Assessment of Negative Symptoms) with univariate statistics corrected for multiple comparisons using false discovery rate. Neuroanatomical differences were examined using voxel-based morphometry (two-sample *t* test SPM12) to identify the brain regions on which the neuroanatomically derived clusters differed. See Supplement section 1.14 for further granular investigation of clinical and inflammatory marker differences between clusters.

### Independent and External Validation

To examine the generalizability of neuroanatomically based clusters, we developed an SVM model using the 280 features on which our HYDRA model was trained (46) to classify patients from the discovery sample into the identified clusters. This SVM was applied to the PRONIA independent replication sample of patients with ROP and ROD (*n* = 404), collected at a different timescale from the discovery sample (May 2016 to February 2019). ComBat was trained on the replication HC group and applied to the replication transdiagnostic patient group to mitigate site effects in the replication dataset. The SVM validation model that was trained on the discovery data was then applied to the replication data.

We externally validated the neuroanatomically based PRONIA clusters using the developed SVM model in three MRI datasets of patients with chronic schizophrenia (Centre for Biomedical Research Excellence [COBRE] and Mind Clinical Imaging Consortium [MCIC]) and chronic depression (Munich [MUC]) in an accelerated longitudinal design framework (Supplement sections 1.9 and 1.10).

### Predictive Utility

**Figure 1.**
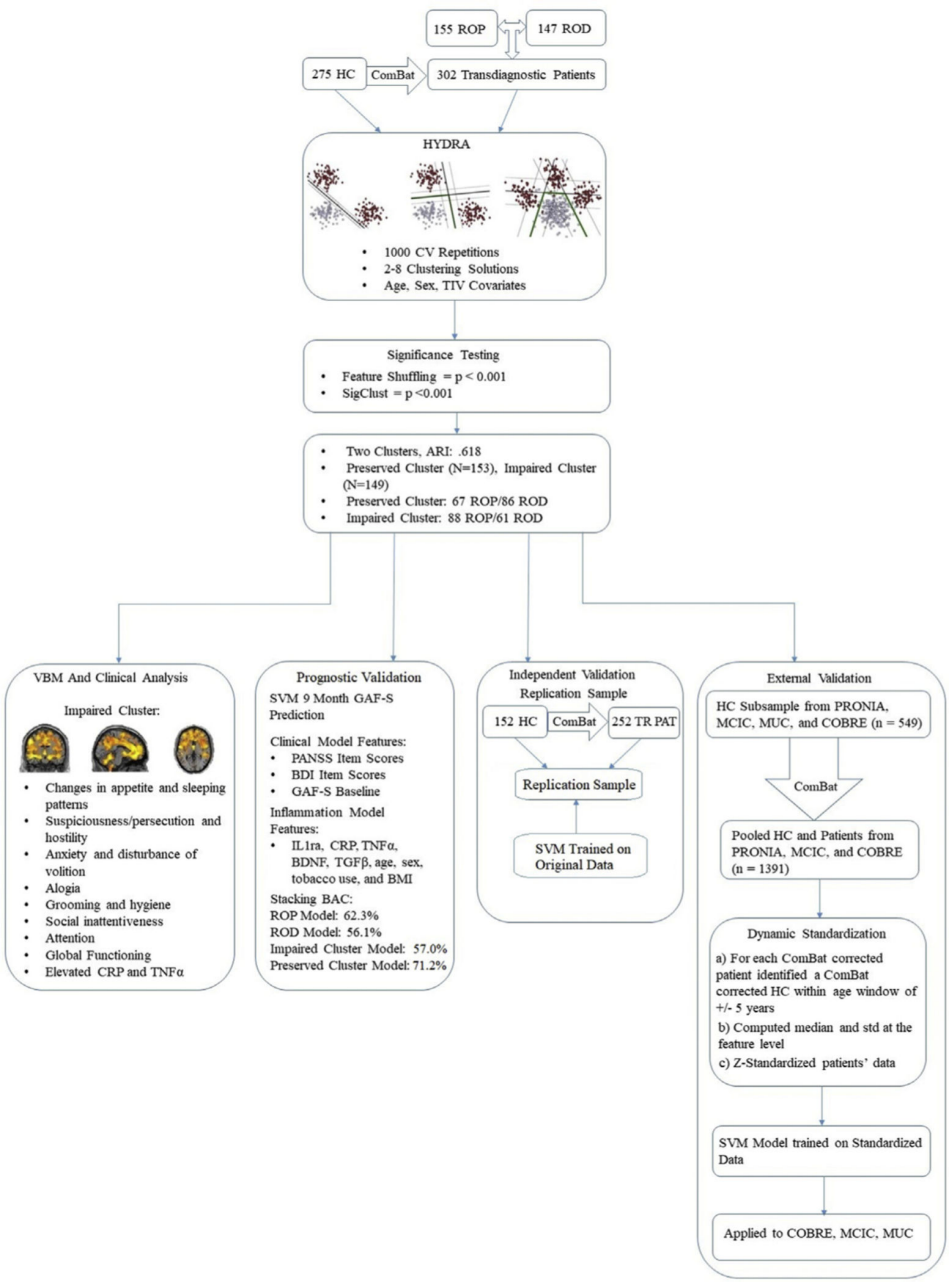
Analysis pipeline overview. This figure provides an overview of the analysis pipeline undertaken in this study. Patients with recent-onset psychosis (ROP) and recent-onset depression (ROD) were combined into one transdiagnostic group. ComBat was trained on healthy control (HC) subjects and applied to the patients to remove site-related variance from the data. HC and patient data were then entered into the HYDRA algorithm with age, sex, and total intracranial volume (TIV) added as covariates. HYDRA was trained using a repeated hold out cross-validation (CV) strategy (i.e., 1000 repetitions with 80% of the data for training in each repetition). The clusters were validated in the PRONIA replication sample and the three external datasets. Identified clusters were assessed for statistical significance and were then analyzed for clinical and voxel-based morphometry (VBM) differences. Furthermore, the predictive utility of the clusters was assessed. BAC, balanced accuracy; BDI, Beck Depression Inventory; BMI, body mass index; BDNF, brain-derived neurotrophic factor; COBRE, Centre for Biomedical Research Excellence; CRP, C-reactive protein; GAF-S, Global Assessment of Functioning-Symptom; IL-1ra, interleukin 1 receptor antagonist; MCIC, Mind Clinical Imaging Consortium; MUC, Munich; PANSS, Positive and Negative Syndrome Scale; PAT, patients; SVM, support-vector machine; TGFβ, transforming growth factor β; TNFα, tumor necrosis factor α; TR, transdiagnostic.

We trained SVM models using symptom and blood-based biomarker data to predict symptom recovery (as defined by a Global Assessment of Functioning-Symptom [GAF-S] score of ≥61) (51) at 9 months. To assess the predictive utility within the neuroanatomically based clusters and within ROP and ROD groups, we trained four different SVM models (one for each different diagnosis of ROP, ROD, cluster 1, and cluster 2) and compared their predictive accuracy in terms of area under the receiver operating characteristic curve, balanced accuracy (BAC), sensitivity, and specificity. Details can be found in Supplement section 1.8. A detailed depiction of the analysis pipeline can be seen in Figure 1.

## RESULTS

### Demographic Information

A total of 155 participants with ROP, 147 patients with ROD, and 275 HC subjects from the discovery sample were included in the HYDRA semi-supervised machine learning analysis. The mean age of the ROP group was 25.3 (SD 5.5) years, the mean age of the ROD group was 25.9 (SD 6.2) years, and the mean age of the HC group was 25.5 (SD 6.4) years. The ROP group consisted of 96 male and 59 female patients, the ROD group had 66 male and 81 female patients, and the HC group had 107 male and 168 female participants. A summary of sociodemographic and clinical information is provided in Table 1. Sociodemographic and clinical information for the PRONIA replication and external validation samples (COBRE, MCIC, and MUC) is provided in Supplement section 1.9.

### HYDRA Semi-supervised Machine Learning Analysis

The optimal clustering solution was two transdiagnostic clusters (cluster 1: *n* = 153, 67 ROP, 86 ROD; cluster 2: *n* = 149, 88 ROP, 61 ROD; adjusted Rand index = 0.618). Patients in cluster 1 had a mean age of 26.2 (6.2) years, and those in cluster 2 had a mean age of 24.9 (5.4) years. There were 78 male and 75 female patients in cluster 1 and 84 male and 65 female patients in cluster 2. The two clusters did not differ in terms of age (*p* = .071), sex distribution (*p* = .358), total intracranial volume (*p* = .144), or medication exposure and differed in terms of original diagnosis distribution (*p* = .008). A sociodemographic and clinical description of the two clusters can be found in Table 1.

### Cluster Statistical Significance

The clusters were statistically significant 1) in terms of whether they would be different than if there was no disease-related variability present (*p* = .010), 2) in terms of whether the disease structures were different (*p* < .001), and 3) in terms of whether the data could be better explained by a single Gaussian distribution (*p* < .001), suggesting that our data could not be explained in terms of a single Gaussian (continuous) distribution assuming a dimensional severity model. Details of the statistical significance tests can be found in Supplement section 1.11.

### Clinical Characteristics Associated With Neuroanatomically Based Clusters

Cluster 2 revealed a more severe symptom presentation than cluster 1, with significantly higher scores in the positive (*t*_287_ = −2.8, *p* = .020), negative (*t*_287_ = −2.2, *p* = .040), and general (*t*_287_ = −2.7, *p* = .010) Positive and Negative Syndrome Scale domains. Patients in cluster 2 had higher negative symptoms in the Scale for the Assessment of Negative Symptoms of affective flattening (*t*_284_ = −2.7, *p* = .010), alogia (*t*_282_ = −3.0, *p* = .020), and attention deficit (*t*_255_ = −2.2, *p* = .040). Patients in cluster 2 also showed worse functioning (Global Functioning-Role) (*t*_291_ = −2.3, *p* = .030). There were no statistically significant differences between the two clusters in terms of neurocognition or blood-based biomarker data in univariate analysis. All *p* values have been false discovery rate corrected (Tables S5–S7). In supplementary multivariate SVM analysis, our neuroanatomically based clusters were separable using cognitive data (BAC = 56.6%, sensitivity = 57.5%, specificity = 55.7%, area under the curve = 0.58, *p* = .01). Patients in cluster 2 mainly exhibited worse cognitive performance in a visual recognition and recall task (Rey–Osterrieth complex figure), and patients in cluster 1 mainly performed worse in verbal memory tasks (Rey Auditory Verbal Learning Test) (Figures S6–S8). The two clusters were also separable by blood-based biomarkers (BAC = 58.7%, sensitivity = 54.9%, specificity = 62.4%, area under the curve = 0.59, *p* = .01), with patients in cluster 2 having elevated levels of CRP and tumor necrosis factor α (Figures S9–S11).

### Voxel-Based Morphometry Analysis of Neuroanatomically Based Clusters

**Figure 2.**
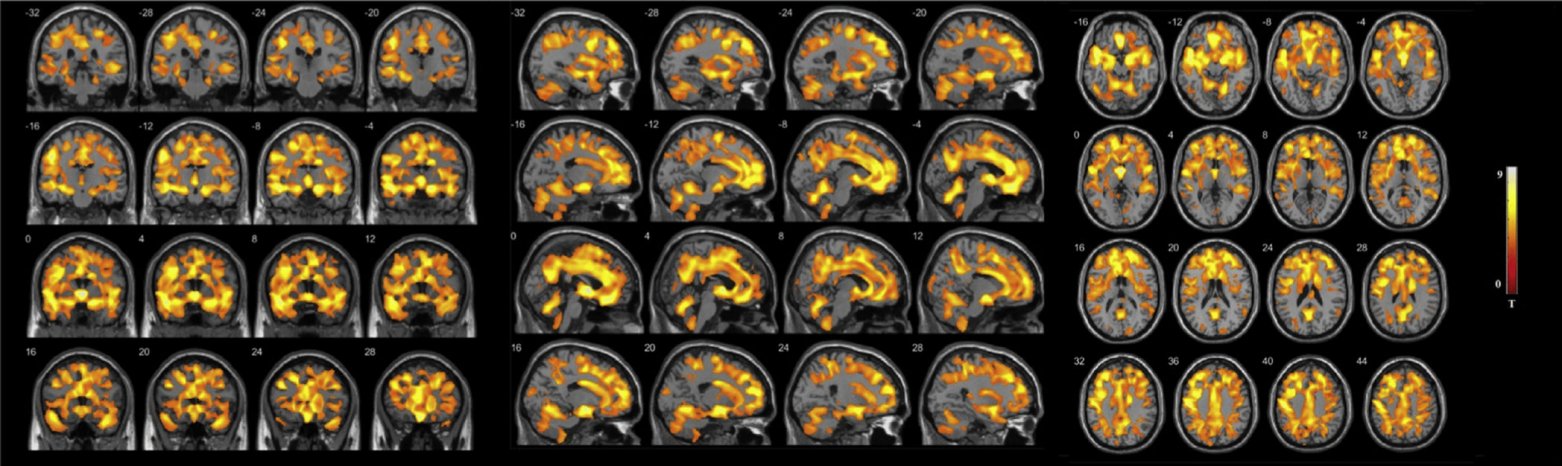
Impaired cluster (cluster 2) gray matter volume reductions compared with the preserved cluster (cluster 1). Gray matter volume reductions are observed in the middle frontal gyrus, superior frontal gyrus, superior temporal gyrus, medial frontal gyrus, cingulate gyrus, right cerebellum, left cerebellum, precuneus, precentral gyrus, inferior frontal gyrus, anterior cingulate, insula, parahippocampal gyrus, left fusiform gyrus, hippocampus, lingual gyrus, amygdala, thalamus, cuneus, middle occipital gyrus, right fusiform gyrus, inferior temporal gyrus, and middle temporal gyrus. Peak voxel Montreal Neurological Institute coordinates can be found in the Supplement (Table S7).

We conducted a voxel-based morphometry analysis for the purpose of demonstrating the brain regions in which the two clusters differed. Here, cluster 2 exhibited widespread GMV loss compared with cluster 1 and HC subjects in areas including the superior temporal gyrus, cingulate gyrus, and thalamus, among others. Cluster 1 revealed increased GMV compared with HC subjects in cerebellar areas. These results can be seen in Figure 2 and in the Supplement (Tables S7 and S8, Figure S2).

### Independent and External Validation

In independent validation, the two-cluster model showed generalizability in the PRONIA replication sample, with patients classified into the two clusters in the replication sample showing similar clinical and neuroanatomical patterns to the ones from the discovery sample (Supplement section 1.18). When externally applied to the MCIC and COBRE (chronic schizophrenia) and MUC (chronic depression) datasets, patients from datasets with a higher mean age and/or longer duration of illness were more often placed in cluster 2, as indicated by negative decision scores. The effects of duration of illness and age were statistically significant ($F_{2,278}$ = 27.88, *p* < .001). Post hoc analyses using the Tukey honestly significant difference post hoc criterion for significance indicated that the mean decision score was significantly lower in the MUC group than in the MCIC group (*p* < .001). Mean decision score differences between the MCIC and COBRE (*p* = .078) groups showed a trend toward statistical significance. The results can be seen in Table 2.

### Prognostic Validation

Within the neuroanatomically based clusters, stacking a blood-based biomarker (IL-1 receptor antagonist, CRP, tumor necrosis factor α, BDNF, and transforming growth factor β) SVM model to a symptom data (baseline Positive and Negative Syndrome Scale, Beck Depression Inventory, and GAF-S individual item scores) SVM model (i.e., a combined model) increased accuracy for predicting symptomatic recovery at 9 months (GAF-S), with BAC of 71.2% for cluster 1% and 57.0% for cluster 2. This outperformed a similar stacked blood-based biomarker and symptom data SVM model predicting GAF-S in ROP and ROD groups (Table 3). A Kruskal-Wallis *H* test showed that there is a statistically significant difference between the outer cross-validation folds (CV2) BAC of the different models (*H*_3_ = 22.9, *p* < .001). Post hoc Mann-Whitney *U* test results can be found in Supplement section 1.13.

## DISCUSSION

In this study, we identified two transdiagnostic clusters across psychosis and depression, using semi-supervised machine learning and neuroanatomical data in a large sample of patients with ROD and ROP. Both clusters contained similar numbers of patients with depression and psychosis; however, they were clinically distinct, with one cluster being characterized by more general and negative symptom loading, functional impairment, and widespread GMV loss (hereafter called the impaired cluster), and one cluster characterized by fewer symptoms, less GMV loss, and less functional impairment but more core depressive symptomatology (hereafter called the preserved cluster). The neuroanatomically based clusters were generalizable to a replication sample and further externally validated in three datasets of patients with chronic illness. Patients with chronic illness, with a higher duration of illness and mean age, were more likely to be classified into the impaired cluster. We were further able to demonstrate that SVM learning models using clinical and blood-based biomarker data to predict symptom remission at 9 months showed a higher accuracy in the neuroanatomically derived clusters compared with traditional diagnostic categories.

The precise etiology of mental illnesses including psychosis and depression, remains elusive despite decades of research, with a stagnation in advance of new pharmacological and psychotherapeutic treatments (52–54). Our results suggest that current diagnostic categories, particularly in early stages of illness, may mask transdiagnostic phenotypes that include an identifiable group with greater impairment and poorer chance of remission across disorders. In our impaired cluster, patients had reduced GMV in areas that have been identified as central to the disease processes of both schizophrenia and depression, such as the superior temporal gyrus, anterior cingulate, insula, and thalamus (55–58). In our analysis, a significant number of patients with depression, who may be perceived as having a less severe illness and better prognostic outlook than patients with psychosis, were ascribed to the impaired phenotype, suggesting that they are on a path toward poor outcome. Conversely, a significant number of patients with psychosis were not assigned to the impaired group and therefore potentially have an identifiable early signature of good prognosis, which was further indicated by the fact that predicting 9-month symptomatic outcomes in that group was more accurate than traditional diagnostic groupings.

Categorical diagnoses have survived because some individuals (specifically those with chronic established illness) do indeed fit within these nosological entities, and more valid solutions remain elusive to date (59). However, within the scope of affective and nonaffective major psychiatric diseases, the Kraepelinian dichotomy of dementia praecox and manic-depressive psychosis has long been challenged. Studies have shown that our understanding of the clinical and neurobiological distinction between disorders may be particularly challenging during early phases of illness (5,25,60,61). The concept of affective disorders as a differential diagnosis for psychosis, particularly in the early years of illness, is waning, with recent research suggesting a central and causal role for depression in the pathogenesis of psychosis and mutual biological underpinnings. This further challenges the distinction between affective and nonaffective pathways to psychosis (25,61–63). Fischer and Carpenter (64) suggest that reducing heterogeneity in syndromes is essential to decisively address the Kraepelinian dichotomy. Despite the fact that dementia praecox does not directly map to nonaffective psychosis, the Verrücktheit (chronic nonaffective psychoses) made distinct in Kraepelin’s first edition (1883) led to the (mis)understanding that schizophrenia was nonaffective (65). The impaired cluster, which contains both patients with schizophrenia and depression, has more cognitive symptoms and a brain signature that is identified in our chronic replication sample. Deficit schizophrenia is a concept introduced over 30 years ago to reduce clinical heterogeneity and suggests the existence of a homogeneous schizophrenia subtype with persistent trait negative symptoms (66). The impaired cluster we identified could be characterized as a transdiagnostic deficit cluster across depression and psychosis due to its higher load of negative symptoms, a previously proposed marker of the deficit syndrome across diagnoses (67). Furthermore, our findings of greater GMV reduction in the impaired cluster corroborate previous research that identified temporal GMV reduction as a marker of very poor outcome (68). Our neuroanatomically derived clusters contained both patients with depression and psychosis in recent onset, replicated in our independent PRONIA sample. This suggests lack of diagnostic hierarchy across depression and psychosis, and that some syndromes may hold equal weight in association with poor outcome regardless of relationship to diagnosis. These results add to the challenge of the separation between affective and nonaffective psychoses, with affective and psychotic diagnostic groups featuring in both clusters, corroborating previous studies that found that high affective symptom scores were equally common in patients with affective and nonaffective psychosis and question the clinical validity of such a distinction (69).

Our results support the common biological susceptibility model of psychiatric disorders and suggest that the biological underpinnings of disease course, at least in depression and psychosis, may be related to transdiagnostic mechanisms, which are potentially hidden by current nosological systems. A similar transdiagnostic model has previously been reported in genomic research, which has shown a certain degree of overlap in biological susceptibility to mental illness across mood and psychotic disorders; evidence of a transdiagnostic biological cause of major psychiatric disorders is evident with the identification of genetic variants that confer a transdiagnostic risk for bipolar disorder, major depressive disorder, and schizophrenia related to the major histocompatibility complex featuring in both schizophrenia and depression genome-wide association studies (70,71). Our finding that elevated proinflammatory cytokines add to predictive accuracy of poor outcome in an impaired phenotype suggests that this genomic immune influence may be ongoing in those on a path to poor outcomes. Schizophrenia GMV deficits in the hippocampus, temporal gyrus, and cerebellum are associated with genetic factors such as *SATB2*, *GABBR2*, and *CACNA1C* (72). A common genetic basis between risk for altered brain structure and neuropsychiatric disorders has been conferred by findings of risk variant enrichment associations with brain structural phenotypes across diagnoses (73). Our results suggest a transdiagnostic cluster of GMV impairment, suggestive of common biological underpinnings for poor outcome across depression and psychosis, with potentially more valid structures than traditional diagnostic categories for use in predicting symptomatic remission.

Heterogeneity and comorbidity may be especially pronounced in the early stages of these disorders; this creates diagnostic uncertainty and difficulties in predicting disease and treatment course (26–30). Our results suggest that a bottomup approach based on neurobiological data may be more reliable in the elucidation of patients with potential for greater impairment and offer a potential future solution for the diagnostic challenges of mental illness. Our external validation findings show that the impaired cluster potentially identifies patients who are on a path to chronic illness from early stages of illness, given that the majority of patients in the external validation sample with chronic illness fell into the same cluster as our impaired group. This has potentially significant clinical implications in terms of personalized treatment and focused recovery interventions. The fact that patients from chronic samples with a higher mean age and illness duration were more likely to be assigned to the impaired cluster could be an indication that our neuroanatomically based clusters identify an accelerated transdiagnostic brain aging effect in recent-onset samples, corroborating previous brain age studies (74,75).

### Strengths and Limitations

This analysis exhibits several strengths including a large dataset with rich clinical, neurocognitive, biomarker, and imaging data from both ROP and ROD groups, independent and external validation, and significance testing of our clustering solutions (e.g., by testing whether the data could be better explained by a Gaussian distribution, which assumes a dimensional severity explanation of the data). Furthermore, the technique we used for the identification of subgroups (HYDRA) offers a solution to issues that are usually associated with clustering based on unsupervised machine learning models that are built on biological data such as the detection of groups that may reflect underlying nuisance variance such as age, sex, body type, and common ancestry (genetics) (76). Nevertheless, our results should be interpreted with caution because there are certain limitations. Due to the nature of our recent-onset sample and using an HC sample as a reference group in the semi-supervised model, there is a risk that the differences between the groups are not as marked as would be seen in more chronic cases. We addressed that limitation by performing permutation tests to robustly assess the significance of the identified clusters. Furthermore, our models were developed in recent-onset patients with a significantly lower mean age than that of our external validation samples. We addressed that limitation by following a robust pipeline that removed age and site effects while retaining disease variance in the data. Although we developed an accelerated longitudinal design with the use of recent-onset and chronic samples and had a 9-month follow-up for prediction of symptom remission, definitive findings would need large longitudinal datasets with repeated measures, such as functional outcome, over many years. Finally, we used only neuroanatomical features to parse neurobiological variance among complex clinical presentations. Psychiatric illness is not a single variable problem, and we have addressed that by examining whether the brainbased clustering solution is reflected in the phenotypic, cognitive, and inflammatory levels. Future studies should consider using multiple biological measures and larger population-level data to encompass the pleiomorphic nature of clinical entities such as depression and psychosis.

## Conclusions

Using semi-supervised machine learning, we were able to identify two neuroanatomically based transdiagnostic clusters. One cluster was characterized by an impaired functional and neurocognitive profile and greater symptomatic loading and GMV loss, while the other cluster was characterized by a more preserved neuroanatomical and reduced symptom signature. Our distinct impaired cluster included patients with depression and psychosis and may provide insight into transdiagnostic etiopathogenetic pathways of chronicity and poor outcome. The identified clusters have been derived in recent-onset samples using structural MRI and could eventually lead to the development of MRI-based prediction and decision-making tools. In external validation, older patients with longer duration of schizophrenia and depression were assigned to the impaired cluster, suggesting a potential identifiable transdiagnostic signature of chronicity and path to poor outcome at the early disease stages. Using clinical and blood-based biomarker data, we were able to predict symptomatic and functional remission more accurately in the derived clusters compared with traditional diagnostic groups. While such challenge to current diagnostic structures will need significant further replication and longer follow-up, identifying a transdiagnostic signature of poor prognosis has the potential to aid new and targeted treatment strategies across early stages of mental disorder.

## Supplementary Material

Supplementary Material

## Figures and Tables

**Table 1.** Sample Sociodemographics

Sample Sociodemographics	ROP Group, *n* = 155	ROD Group, *n* = 147	ROP vs. ROD		Cluster 1 (Preserved), *n* = 153	Cluster 2 (Impaired), *n* = 149	Cluster 1 vs. Cluster 2		HC, *n* = 275	HC vs. Impaired Cluster		HC vs. Preserved Cluster	
			$t/\chi^{2}$	*p* Value			$t/\chi^{2}$	*p* Value		$t/\chi^{2}$	*p* Value	$t/\chi^{2}$	*p* Value
Original Diagnostic Group, ROP/ROD, *n* (%)	-	-	-	-	67/86 (43.2%/58.5%)	88/61 (56.8%/41.5%)	${\chi^{2}}_{1}$ = 7.04	.008	-	-	-	-	-
Age, Years, Mean (SD)	25.3 (5.5)	25.9 (6.2)	$t_{300}$ = −0.879	.380	26.2 (6.2)	24.9 (5.4)	$t_{300}$ = 1.81	.071	25.5 (6.4)	$t_{422}$ = 0.887	.375	$t_{426}$ = −1.035	.301
Sex, Female/Male, *n*	59/96	81/66	${\chi^{2}}_{1}$ = 8.8	.003	75/78	65/84	${\chi^{2}}_{1}$ = 0.88	.358	168/107	${\chi^{2}}_{1}$ = 11.9	.001	${\chi^{2}}_{1}$ = 5.8	.016
Total Intracranial Volume, Mean (SD)	1531.6 (141.9)	1500.6 (144.3)	$t_{300}$ = 1.87	.061	1504.6 (144.0)	1528.7 (142.8)	$t_{300}$ = −1.46	.144	1518.5 (140.8)	$t_{422}$ = −0.708	.481	$t_{426}$ = 0.975	.330
Medication, Mean Cumulative Sum (SD)													
CPZE	-	-	-	-	5122.7 (16,501.2)	11,191.7 (52,988.6)	$t_{300}$ = −1.24	.214	-	-	-	-	-
OLAE	-	-	-	-	390.5 (1780.0)	173.9 (551.4)	$t_{300}$ = −1.32	.187	-	-	-	-	-
SSRIE	-	-	-	-	3095.7 (10,409.5)	2504.3 (7975.8)	$t_{300}$ = 0.510	.610	-	-	-	-	-
BENZOE	-	-	-	-	282.8 (1031.5)	578.6 (3625.2)	$t_{300}$ = −0.888	.375	-	-	-	-	-
SCID Diagnosis, *n* (%)													
Schizophrenia	63 (40.6%)	0 (0%)	-	-	22 (14.4%)	41 (27.5%)	-	-	-	-	-	-	-
Schizophreniform disorder	12 (7.7%)	0 (0%)	-	-	3 (2.0%)	9 (6.0%)	-	-	-	-	-	-	-
Schizoaffective disorder	8 (5.2%)	0 (0%)	-	-	4 (2.6%)	4 (2.7%)	-	-	-	-	-	-	-
Delusional disorder	8 (5.2%)	0 (0%)	-	-	7 (4.6%)	1 (0.7%)	-	-	-	-	-	-	-
Psychotic disorder NOS	22 (14.2%)	0 (0%)	-	-	11 (7.2%)	11 (7.4%)	-	-	-	-	-	-	-
Major depressive disorder	13 (8.4%)	140 (95.2%)	-	-	88 (57.5%)	65 (43.6%)	-	-	-	-	-	-	-
Bipolar disorder I	9 (5.8%)	0 (0%)	-	-	4 (2.6%)	5 (3.4%)	-	-	-	-	-	-	-
Other	20 (12.9%)	7 (4.8%)	-	-	14 (9.1%)	13 (8.7%)	-	-	-	-	-	-	-
PANSS Positive Score, Mean (SD)	17.5 (6.3)	7.6 (1.2)	300 = 18.25	<.001	11.5 (5.8)	13.1 (7.4)	$t_{300}$ = −2.83	.02	-	-	-	-	-
PANSS Negative Score, Mean (SD)	16.4 (7.9)	12.2 (4.7)	$t_{300}$ = 5.43	<.001	13.5 (6.3)	15.2 (7.2)	$t_{300}$ = −2.21	.04	-	-	-	-	-
PANSS General Score, Mean (SD)	35.7 (11.6)	27.1 (6.5)	$t_{300}$ = 7.99	<.001	29.8 (8.2)	33.0 (11.4)	$t_{300}$ = −2.71	.01	-	-	-	-	-

BENZOE, benzodiazepine equivalent; CPZE, chlorpromazine equivalent; HC, healthy control; NOS, not otherwise specified; OLAE, olanzapine equivalent; PANSS, Positive and Negative Syndrome Scale; ROD, recent-onset depression; ROP, recent-onset psychosis; SCID, Structured Clinical Interview for DSM Disorders; SSRIE, selective serotonin reuptake inhibitor equivalent.

**Table 2.** External Validation Results

Results	COBRE, *n* = 71	MCIC, *n* = 107	MUC, *n* = 103
Diagnosis	Schizophrenia	Schizophrenia	Depression
Age, Years	38.1 (13.9)	34.5 (11.1)	42.1 (11.9)
Duration of Illness, Years	16.8 (12.9)	10.9 (10.9)	5.8 (7.7)
Decision Score	−0.04 (0.63)	0.15 (0.71)	−0.47 (0.48)

Values are presented as mean (SD). Decision scores reflect mean distance of patients from the hyperplane separating the two clusters. Positive decision scores indicate assignment to cluster 1 (preserved cluster) and negative decision scores indicate assignment to cluster 2 (impaired cluster) ($F_{2,278}$ = 27.88, *p* < .001).

COBRE, Centre for Biomedical Research Excellence; MCIC, Mind Clinical Imaging Consortium; MUC, Munich.

**Table 3.** SVM Models Predicting 9-Month GAF-S Remission

SVM 9-Month Models	True Positive, *n*	True Negative, *n*	False Positive, *n*	False Negative, *n*	Correct Classification Rate		Balanced Accuracy, %	Positive Predictive Value, %	Negative Predictive Value, %	AUC	Model *p* Value
					Unremitted, %	Remitted, %					
Stacked ROP Model	20	33	19	29	40.8%	63.5%	52.1%	51.3%	53.2%	0.56	.38
Stacked ROD Model	53	11	13	26	67.1%	45.8%	56.5%	80.3%	29.7%	0.54	.17
Stacked Preserved Cluster Model	19	54	11	13	59.4%	83.1%	71.2%	63.3%	80.6%	0.72	.07
Stacked Impaired Cluster	35	25	16	31	53.0%	61.0%	57.0%	68.6%	44.6%	0.58	.18

*H*_3_ = 22.9, *p* < .001.

AUC, area under the curve; GAF-S, Global Assessment of Functioning-Symptom; SVM, support-vector machine; ROD, recent-onset depression; ROP, recent-onset psychosis.

**Table:** KEY RESOURCES TABLE

Resource Type	Specific Reagent or Resource	Source or Reference	Identifiers	Additional Information
Add additional rows as needed for each resource type	Include species and sex when applicable.	Include name of manufacturer, company, repository, individual, or research lab. Include PMID or DOI for references; use “this paper” if new.	Include catalog numbers, stock numbers, database IDs or accession numbers, and/or RRIDs. RRIDs are highly encouraged; search for RRIDs at https://scicrunch.org/resources.	Include any additional information or notes if necessary.
Antibody	N/A			
Bacterial or Viral Strain	N/A			
Biological Sample	Human Blood Samples	Luminex platform (Bio-Plex 200 system with Bio-Plex Manager software)		
Cell Line	N/A			
Chemical Compound or Drug	N/A			
Commercial Assay Or Kit	N/A			
Deposited Data; Public Database	N/A			
Genetic Reagent	N/A			
Organism/Strain	N/A			
Peptide, Recombinant Protein	N/A			
Recombinant DNA	N/A			
Sequence-Based Reagent	N/A			
Software; Algorithm	HYDRA	https://github.com/anbai106/mlni		
Software; Algorithm	Neurominer	https://github.com/neurominer-git/NeuroMiner-1		
Software; Algorithm	SigClust	https://cran.r-project.org/web/packages/sigclust/sigclust.pdf		
Software; Algorithm	ComBat	https://github.com/Jfortin1/ComBatHarmonization		
Transfected Construct	N/A			
Other	N/A			
