# Supplementary material for: Neurobiologically Based Stratification of Recent-Onset Depression and Psychosis: Identification of Two Distinct Transdiagnostic Phenotypes
Source: Biol Psychiatry. Author manuscript; Available in PMC 2023 Apr 25. (PMC10128104; doi:10.1016/j.biopsych.2022.03.021)
Supplement: Supplementary Material [file NIHMS1883256-supplement-Supplementary_Material.pdf]

Neurobiologically Based Stratification of Recent-Onset Depression and Psychosis: Identification of Two Distinct Transdiagnostic Phenotypes

Supplement

Supplementary Methods and Results

1. Supplementary Methods

1.1. PRONIA recruitment infrastructure

The 981 study participants (155 individuals with ROP, 147 individuals with ROD, and 275 HC from the discovery sample as well as 129 individuals with ROP, 123 individuals with ROD, and 152 HC from the replication sample) analyzed in the present study were recruited following a standardized recruitment and ascertainment protocol (see **Figure S1** and **Table S3**). The observational study protocol involved follow-up examinations every three months after the index ascertainment and was implemented by the following 7 PRONIA sites:

Table S1: Characteristics of the recruiting institutions in the PRONIA consortium (Directly reproduced from a previous study[1]).

PRONIA Site	Institution Name	Country	Type of Service	Catchment Population	Screening population / year
Munich	Department of Psychiatry and Psychotherapy, Ludwig-Maximilian-University Munich	DE	Academic outpatient services including specialized service for early recognition of psychosis; tertiary care academic hospital	1,200,000	700
Basel	Department of Psychiatry and Psychotherapy, University of Basel	CH	Academic inpatient and outpatient services including specialized service for early recognition and intervention of psychosis; tertiary care academic hospital	500,000	200
Milan Niguarda	Department of Pathophysiology and Transplantation, University of Milan. Four recruitment hospitals: Niguarda, Policlinico, San Paolo, Villa San Benedetto Menni in Albese con Cassano	IT	Psychiatric outpatient services including specialized services for early recognition of psychosis and persons at high risk; Academic hospital, providing psychiatric inpatient services, psychiatric outpatient services and local services;	600,000	1,000
Cologne	Department of Psychiatry and Psychotherapy, University of Cologne	DE	Academic outpatient services including specialized service for early recognition of psychosis; tertiary care academic hospital	1,000,000	600
Birmingham	The University of Birmingham	UK	Academic specialised Early Intervention Service for Psychosis covering Birmingham and Solihull. Community and Inpatient	1,200,000	800
Turku	Department of Psychiatry, University of Turku	FI	Psychiatric outpatient and hospital services responsible for treatment of psychiatric patients in their catchment	284,000	2,300

			areas in the South-Western Finland.		
Udine	Department of Psychiatry, University of Udine	IT	Psychiatic outpatient services, academic hospital and local services. Tertiary care neuropsychiatric service	600,000	500

Upon study enrolment, the participants were pseudonymized twice, locally at each site and centrally at the level of the PRONIA portal. The PRONIA portal consists of a multi-user database hosting the clinical and neurocognitive information, and defaced MR images obtained from the study participants. The data are organized into digital questionnaires, visits, and cases. The portal provides the case managers with a controlled web-based interface to enter and upload the different data into the respective questionnaires. Furthermore, the PRONIA consortium has implemented a PRONIA@home mobile device interface that allows the study participants to securely log into the portal and fill out the self-rating questionnaires of given visit. Upon completion of the data entry across all questionnaires of given visit, the data are checked by an automatic quality control procedure which executes approximately 1600 data integrity and dependency rules. These rules include (1) basic checking of missing data and data ranges, (2) checking of dependency within one questionnaire, (3) dependencies between two questionnaires within one visit, and (4) dependencies between two consecutive visits (such as consistency of dates). Detected errors are fed back to the respective case managers allowing for a manual correction of the respective issues. This process is re-iterated until the quality of the clinical questionnaires in the given visit is sufficient for the entire visit to be locked.

1.2. PRONIA study design and examination instruments

A comprehensive battery of ascertainment tools was used within a longitudinal observational study design to generate a multi-modal phenotypic profile of each study participant (see **Figure S1** and **Table S2**). The clinical part of the battery compiled questionnaires that capture sociodemographic, somatic, environmental, diagnostic, psychopathological, functional and quality-of-life related variables in the PRONIA study population. This battery was complemented by multi-domain neurocognitive and neuroimaging examinations as well as blood sampling for later genetic characterization, which were carried out at the baseline and 9-month follow-up timepoints.

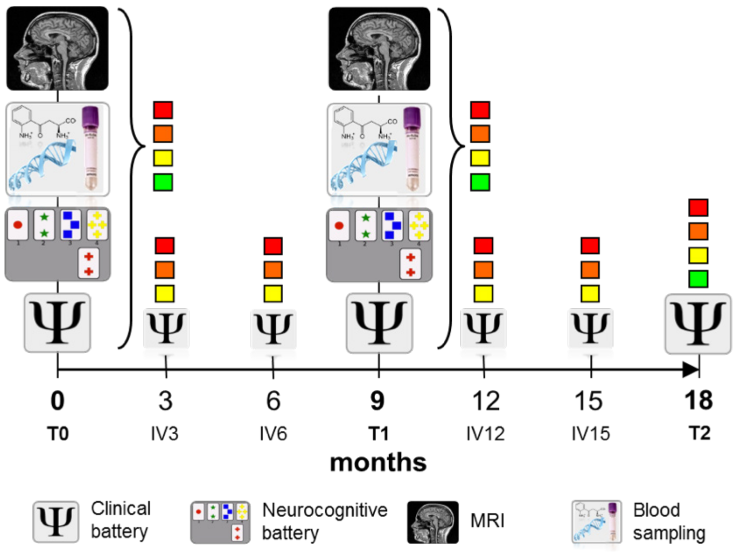

Figure S1: Observational study design of PRONIA (Directly reproduced from a previous study[1]). Colored boxed indicate type of assessment / visits conducted in each of the study groups: Healthy controls (green), patients with recent-onset depression (yellow), persons with a clinical high-risk for psychosis (orange), patients with recent-onset psychosis (red).

Table S2: Clinical and neurocognitive examinations performed in the CHR, ROD, ROP, and HC groups during the 18-month follow-up period of the study (Directly reproduced from a previous study[1]). Clinical assessment types: *OR* Observer-based rating instrument, *SR* Self-rating-based instrument. **Examination timepoints:** *T0* Baseline examination, *IV3/IV6/IV12/IV15* 3, 6, 12, 15-month examinations conducted only in the clinical study participants, *T1* 9-month examination, *T2* 18-month follow-up examination. **Observer-based instruments:** CAARMS Comprehensive Assessment of the At-Risk Mental States[2], *CHR Criteria* Clinical High-Risk criteria summary questionnaire, *FROGS* Functional Remission in General Schizophrenia[3], *GAF* Global Assessment of Functioning, *GF:S/R* Global Functioning: Social / Role[4], *PANSS* Positive and Negative Symptom Scale[5], *PAS* Premorbid Adjustment Scale[6], *SANS* Scale for the Assessment of Negative Symptoms[7], *SCID-IV Screening/Summary* Structured Clinical Interview for DSM-IV[8], *SIPS* Standardized Interview for the Assessment of Prodromal Symptoms (modified version 5.0)[9], *SPI-A [COGDIS/COPER]* Schizophrenia Proneness Instrument [Cognitive disturbances (COGDIS) / Cognitive-Perceptual (COPER) disturbances][10], Transition Criteria Interval questionnaire for the assessment of transition criteria, *UHR - Schizotypy, Genetic Risk* Interview for the Assessment of Schizotypal personality traits, and familial risk for psychosis. **Self-rating instruments:** *BDI-II* Beck Depression Inventory II[11], *CISS-24* Coping

Inventory for Stressful Situations – 24 items[12], *CTQ* Childhood Trauma Questionnaire[13], *EHI-SR* Edinburgh Handedness Inventory – Short Version[14], *EDS* Everyday Discrimination Scale – Modified Version[15], *LEE* Level of Expressed Emotions[16], *MSPSS* the Multidimensional Scale for Perceived Social Support[17], *NEO-FFI* NEO Five Factor Inventory of Personality Traits[18], *RSA* Resilience Scale for Adults[19], *SPIN* Social Phobia Inventory[20], *WHO-QOL-BREF* WHO Quality of Life Questionnaire-Brief Version[21]. Neurocognitive tests: *CPT-IP* Continuous-Performance Test-Identical Pairs (adapted tablet version)[22], *DANVA* Diagnostic Analysis of Non-Verbal Accuracy 2 (adapted tablet version)[23], *DS* Auditory Digit Span (Forward/Backward) adapted from the PEBL battery, *DSST* Digit-Symbol-Substitution Test from the BACS battery, *ROCF* Rey-Osterrieth complex figure[24], *SAT* Salience Attribution Task (adapted version)[25], *SOPT* self-ordered pointing task (adapted version)[26], *TMT-A/-B* Trail-Making Test A and B[27], *VF* phonemic/semantic verbal fluency test.

**Instrument**	**Form**	**Screening**		**T0**		**IV3**	**IV6**	**T1**		**IV12**	**IV15**	**T2**	
		PAT	HC	PAT	HC	PAT	PAT	PAT	HC	PAT	PAT	PAT	HC
General Data	OR	X	X					X	X			X	X
Reasons for Referral	OR	X											
Treatment Documentation	OR	X	X			X	X	X	X	X	X	X	X
Somatic state and Health History	OR	X	X					X	X			X	X
SPI-A COGDIS/COPER	OR	X	X			X	X	X	X	X	X	X	X
SIPS positive symptoms	OR	X	X			X		X	X			X	X
CAARMS	OR	X	X			X		X	X			X	X
GAF	OR	X	X			X		X	X			X	X
UHR – Schizotypy, Genetic Risk	OR	X	X			X		X	X			X	X
CHR Criteria	OR	X	X					X	X			X	X
Transition Criteria	OR					X	X			X	X		
SCID-IV Screening	OR	X	X					X	X			X	X
SCID-IV Summary	OR	X	X					X	X			X	X
Demographic and Biographic Data	OR			X	X			X	X			X	X
PAS	OR			X	X			X				X	
SPI-A	OR			X	X			X				X	
SIPS negative, disorganized and general symptoms	OR			X	X			X				X	
PANSS	OR			X		X	X	X		X	X	X	
SANS	OR			X				X				X	
Chart of Life Events	OR			X	X	X	X	X	X	X	X	X	X
FROGS	OR			X				X				X	
GF: Social & Role	OR			X	X	X	X	X	X	X	X	X	X
Prognostic evaluation	OR			X				X				X	
MSPSS	SR			X	X			X	X			X	X
RSA	SR			X	X			X	X			X	X
CISS 24	SR			X	X			X	X			X	X
SPIN	SR			X	X			X	X			X	X
BDI-II	SR			X	X	X	X	X	X	X	X	X	X
WHO-QOL-BREF	SR			X	X			X	X			X	X
EHI-SR	SR			X	X								
LEE	SR			X	X			X	X			X	X
Wisconsin Scales	SR			X	X								
EDS	SR			X	X								
Bullying Scale T0	SR			X	X								
CTQ	SR			X	X								
NEO-FFI	SR			X	X								
DS backward (BACS)	NPT			X	X			X	X				
DS forward (BACS)	NPT			X	X			X	X				
CPT-IP (BACS)	NPT			X	X			X	X				
DANVA	NPT			X	X			X	X				
DSST	NPT			X	X			X	X				
RAVLT	NPT			X	X			X	X				
ROCF	NPT			X	X			X	X				
SAT	NPT			X	X			X	X				
SOPT	NPT			X	X			X	X				
TMT-A	NPT			X	X			X	X				
TMT-B	NPT			X	X			X	X				
VF phonetic	NPT			X	X			X	X				
VF semantic	NPT			X	X			X	X				
WAIS-III	NPT			X	X			X	X				

1.3. MRI harmonization and data acquisition

When setting up the PRONIA study, we decided to generate a MRI database that would represent the MR scanner sequence heterogeneity encountered in clinical real-world. The aim of this strategy was to strengthen the generalizability and clinical applicability of the predictive models developed by our machine learning analyses. Thus, we agreed on a minimal harmonization protocol that required the PRONIA sites to only (1) acquire isotropic or nearly isotropic voxel sizes of preferably 1 mm resolution, (2) set the Field Of View (FOV) parameters accordingly to guarantee the full 3D coverage of the brain including all parts of the cerebellum, and (3) define the relaxation time (TR) and echo time (TE) as well as other imaging parameters in a way that would maximize the contrast between cortical ribbon and the white matter and enhance the signal-to-noise ratio in the images. The sMRI images of six healthy travelling volunteers who were scanned at all sites with the same parameters, were also analysed as part of a calibration study. At every site all the images were visually inspected, automatically defaced and anonymized using an in-house Freesurfer-based script before the data was centralized. **Table S3** lists the parameters defining the structural MR sequences used to examine in the PRONIA discovery sample participants.

Table S3: MR scanner systems and structural MRI sequence parameters used at the respective PRONIA sites (Directly reproduced from a previous study[1]). .

PRONIA Site	Model	Field Strength	Coil Channels	Flip Angle	TR [ms]	TE [ms]	Voxel Size [mm]	FOV	Slice Number
Munich	Philips Ingenia	3T	32	8	9.5	5.5	0.97 x 0.97 x 1.0	250 x 250	190
Milan Niguarda	Philips Achieva Intera	1.5T	8	12	Shortest (8.1)	Shortest (3.7)	0.93 x 0.93 x 1.0	240 x 240	170
Basel	SIEMENS Verio	3T	12	8	2000	3.4	1.0 x 1.0 x 1.0	256 x 256	176
Cologne	Philips Achieva	3T	8	8	9.5	5.5	0.97 x 0.97 x 1.0	250 x 250	190
Birmingham	Philips Achieva	3T	32	8	8.4	3.8	1.0 x 1.0 x 1.0	288 x 288	175
Turku	Philips Ingenuity	3T	32	7	8.1	3.7	1.0 x 1.0 x 1.0	256 x 256	176
Udine	Philips Achieva	3T	8	12	Shortest (8.1)	Shortest (3.7)	0.93 x 0.93 x 1.0	240 x 240	170

1.4. MRI processing pipeline

The manual of the CAT12 toolbox (http://www.neuro.uni-jena.de/cat12/CAT12-Manual.pdf) details the processing steps applied to the structural images. These steps consist of:

(1) A 1st denoising step based on Spatially Adaptive Non-Local Means (SANLM) filtering[28].

(2) An Adaptive Maximum A Posteriori (AMAP) segmentation technique, which models local variations of intensity distributions as slowly varying spatial functions and thus achieves a homogeneous segmentation across cortical and subcortical structures[29].

(3) A 2nd denoising step using Markov Random Field approach which incorporates spatial prior information of adjacent voxels into the segmentation estimation generated by AMAP[29].

(4) A Local Adaptive Segmentation (LAS) step, which adjusts the images for white matter (WM) inhomogeneities and varying gray matter (GM) intensities caused by differing iron content in e.g. cortical and subcortical structures. The LAS step is carried out before the final AMAP segmentation.

(5) A Partial Volume Segmentation algorithm that is capable of modeling tissues with intensities between GM and WM, as well as GM and cerebrospinal fluid (CSF) and is applied to the AMAP-generated tissue segments.

(6) A high-dimensional DARTEL registration of the image to a MNI-template generated from the MRI data of 555 healthy controls in the IXI database (http://www.braindevelopment.org).

(7) The GM maps were then multiplied with the Jacobian determinants that were obtained during registration in order to produce GM volume maps.

(8) The Quality Assurance framework of CAT12 was used to check the quality of the GMV maps.

1.5 ROP and ROD Inclusion Criteria

The general inclusion criteria for the study were: (1) age between 15 and 40 years, (2) sufficient language skills for participation, (3) capacity to provide informed consent/assent. General exclusion criteria were: (1) an IQ below 70, (2) current or past head trauma with loss of consciousness (> 5 minutes), (3) current or past known neurological or somatic disorders potentially affecting structure or functioning of the brain, (4) current or past alcohol dependence, (5) polysubstance dependence within the past six months, and (6) any medical indication against MRI. Recent onset psychosis (ROP) and recent onset depression (ROD) inclusion criteria can be found in the supplement (1.5). ROP participants had to meet the following criteria: 1) DSM-IV-TR (Diagnostic and Statistical Manual of Mental Disorders, Text Revision) (43) affective or non-affective psychotic episode (lifetime), 2) criteria for DSM-IV-TR affective or nonaffective psychotic episode fulfilled within past 3 months and 3) onset of psychosis within past 24 months. ROD patients had to meet the following criteria: 1) DSM-IV-TR major depressive episode (lifetime), 2) major depressive disorder criteria fulfilled within past three months and 3) duration of first depressive episode no longer than 24 months. Specific ROD exclusion criteria were: (1) a previous episode of DSM-IV-TR major depression prior to the current or recent episode, (2) a duration of the current episode exceeding 24 months, (3) antipsychotic medication exceeding 30 days (cumulative in the past 24 months) with a daily dose rate at or above minimum dosage of the "first episode psychosis" range of DGPPN S3 guidelines[31], and (4) any antipsychotic medication within the past three months prior to baseline assessments at or above minimum dosage of the "first episode psychosis' range of DGPPN S3[31] (See Table S4).

Table S4: DGPPN S3 Guidelines for the treatment of first-episode psychosis and schizophrenia *(translated English version of Table 4.1 stated in the short version of the guideline manual available in https://www.dgppn.de/_Resources/Persistent/a6e04aa47e146de9e159fd2ca1e6987853a055d7/S3_Schizo_Kurzversion.pdf)* ***(Directly reproduced from a previous study[1]).*** *Candidate CHR and ROD patients were excluded if they had received antipsychotic medication (1) for more than 30 cumulative days at or above the minimum target dosage threshold for the treatment of first-episode psychosis, or (2) within the past 3 months before psychopathological baseline assessments at or above the minimum target dosage threshold for the treatment of first-episode psychosis. Abbreviations:* DI *dosage interval,* [2]*maximum recommended dosage according to prescribing information.*

Substance	Recommended starting dosage (mg/d)	DI'	Target dosage first-episode psychosis (mg/d)	Target dosage relapsing schizophrenia (mg/d)	Maximum dosage recommended (mg/d)[2]
**Atypical Antipsychotics**					
Amisulpride	200	(1)-2	100-300	400-800	1200
Aripiprazole	(10)-15	1	15-(30)	15-30	30
Olanzapine	5-10	1	5-15	5-20	20
Quetiapine	50	2	300-600	400-750	750
Risperidone	2	1-2	1-4	3-6-(10)	16
Ziprasidone	40	2	40-80	80-160	160
**Typical Antipsychotics**					
Fluphenazine	0.4-10	2-3	2.4-10	10-20	20-(40)
Flupentixole	2-10	1-3	2-10	10-60	60
Haloperidole	1-10	(1)-2	1-4	3-15	100
Perazine	50-150	1-2	100-300	200-600	1000
Perphenazine	4-24	1-3	6-36	12-42	56
Pimozide	1-4	2	1-4	2-12	16
Zotepine	25-50	2-(4)	50-150	75-150	450
Zuclopenthixole	2-50	1-3	2-10	25-50	75

1.6 HYDRA

HYDRA is a hybrid between unsupervised clustering and supervised classification methods; it can simultaneously fit maximum margin classification boundaries and elucidate disease subtypes, which is not possible with unsupervised clustering methods or non-linear kernel classifiers. HYDRA investigates different directions of deviation from normal anatomy (as

dictated by a healthy control reference sample) with no regard for their magnitude. Patients are grouped into clusters based on the brain regions which are different from the healthy controls and not based on the magnitude by which those regions are different. A number of unique hyperplanes is chosen with the application of Determinental Point Processes (DPP) which is technique that helps derive the most diverse samples possible. This ensures that identified regions reflect biomarkers that are unique rather than repeated ones with varying magnitudes and helps ensure clusters of patients that are reflective of disease related variability.

1.7 Blood-Based Biomarker Data

Serum: Following a strict protocol, a 9 ml blood sample was collected from consenting participants in S-monovette serum tubes for isolation of serum. Blood samples were drawn in the morning after 12 hours fasting where possible. Samples were inverted 5-6 times and allowed to stand at room temperature for 30 minutes to coagulate. After centrifugation for 10 minutes at 2750g, serum was pipetted in 8 300ul aliquots in cryo vials and stored at -80oC until analysis.

Blood: at the same sampling time, two 9ml fasting whole blood samples were collected in EDTA tubes and stored at -80oC until genomic analysis.

Serum Peripheral Inflammatory Marker Assays:

Samples were transferred to the University of Birmingham Barnes Laboratory in March 2019. There were no freeze-thaw cycles before analysis. Of samples included in inflammatory marker analysis, 83% were taken before 12 noon and 76% were fasting. 95% of participants were in a sitting position when the blood was drawn. The average length of serum aliquot sample storage until analysis was 48.78 months (median = 45 months). All samples were assayed blind to subject information.

An aliquot from each sample was centrifuged (15 min, 15k x g, 4°C) before analysis using the Luminex platform (Bio-Plex 200 system with Bio-Plex Manager software) using a commercial multiplex kit for IFNg, IL-1b, IL-1ra, IL-2, IL-4, IL-6, S100B and TNFa (Bio-Techne), and commercial singleplex kits for CRP (Bio-Techne), BDNF (Bio-Techne) and TGFb1 (Bio-Techne) following the manufacturer's instructions.

1.8 Independent Validation

We followed the exact same procedure as described in main manuscript in order to develop a model in the replication sample. In order to mitigate site effects, prior to applying HYDRA, the R version of the ComBat harmonization technique was employed (https://github.com/Jfortin1/ComBatHarmonization). To further ensure that disease variance would be retained and that scanner variance would be disentangled from disease variance, ComBat was trained on the replication healthy controls and then ~~applied~~ the estimates that were derived were applied to the replication patients. We used whole brain measures derived from CAT12 from 280 Regions of the neuromorphometrics atlas parcellation from 404 participants with Recent Onset Psychosis (ROP) and Recent Onset Depression (ROD) and Healthy Controls (HC) from the replication sample of the PRONIA study. ROP patients and ROD patients were grouped together into one patient group. HYDRA was trained using a 1000-fold cross-validation method using age, sex, and Total Intracranial Volume (TIV) as covariates, and requesting 2 to 8 clustering solutions. Adjusted Rand Index (ARI) was used to measure cluster stability. The statistical significance of clusters was assessed in two ways. We assessed whether the obtained subgroups were different to what we would find if there was no disease present by comparing the observed ARI to a null distribution of ARI. We also assessed whether clusters really are present in the dataset or if there is a single Gaussian distribution by employing SigClust [32](https://github.com/pkimes/sigclust2). Using the discovery data (280 neuromorphometrics features) we then trained a SVM model which classified patients into

either the impaired or preserved cluster in order to develop a tool that allows us to apply our clustering solution to external datasets without the need to rerun HYDRA on the original (discovery) data. We then applied that model to the replication sample data. Details of the machine learning pipeline are described in 1.12.

1.9 External Validation

COBRE Dataset

The Center for Biomedical Research Excellence (COBRE) is contributing raw anatomical and functional MR data from 72 patients with Schizophrenia and 75 healthy controls (ages ranging from 18 to 65 in each group). All subjects were screened and excluded if they had; history of neurological disorder, history of intellectual disability, history of severe head trauma with more than 5 minutes loss of consciousness, history of substance abuse or dependence within the last 12 months. Diagnostic information was collected using the Structured Clinical Interview used for DSM Disorders (SCID).

A multi-echo MPRAGE (MEMPR) sequence was used with the following parameters: TR/TE/TI = 2530/[1.64, 3.5, 5.36, 7.22, 9.08]/900 ms, flip angle = 7°, FOV = 256x256 mm, Slab thickness = 176 mm, Matrix = 256x256x176, Voxel size =1x1x1 mm, Number of echos = 5, Pixel bandwidth =650 Hz, Total scan time = 6 min. With 5 echoes, the TR, TI and time to encode partitions for the MEMPR are similar to that of a conventional MPRAGE, resulting in similar GM/WM/CSF contrast.

Rest data was collected with single-shot full k-space echo-planar imaging (EPI) with ramp sampling correction using the intercomissural line (AC-PC) as a reference (TR: 2 s, TE: 29 ms, matrix size: 64x64, 32 slices, voxel size: 3x3x4 mm3).

Slice Acquisition Order:

Rest scan - collected in the Axial plane - series ascending - multi slice mode - interleaved

MPRAGE - collected in the Sag plane - series interleaved - multi slice mode - single shot

The following data are released for every participant:

Resting fMRI

Anatomical MRI

Phenotypic data for every participant including: gender, age, handedness and diagnostic information.

MCIC Dataset

Details about the MCIC dataset are described in a prior publication[33].

Depression Dataset

Details about the MUC dataset are described in a prior publication[34].

1.10 External Validation Pipeline

The COBRE dataset has the highest duration of illness in years (M=16.8. SD=12.9), followed by MCIC (M=10.9, SD=10.9), and MUC (M=5.8, SD=7.7). The MUC dataset had the oldest subjects (M= 42.1, SD=11.9), followed by COBRE (M=38.1, SD=13.9), and MCIC (M=34.5, SD=11.1). Due to the significant age differences between the PRONIA recent onset samples and the older external validation samples we followed a robust pipeline that removed the age and site effects while retaining the disease variance in the data (see supplementary methods 1.10). Due to the significant age differences between the PRONIA recent onset samples and the older external validation samples HYDRA was not performed in the external datasets and we followed a robust pipeline that removed the age and site effects while retaining the disease variance in the data. First, we identified a HC subsample across different sites in the PRONIA

and external validation datasets which was age and sex matched and had a specific age range (no older than 40 years of age). We then pooled the HC and patients from the PRONIA, COBRE, MCIC, and MUC datasets. Then, we trained ComBat on the identified HC subsample and applied it to the pooled HC and patients. Afterwards, we used the full ComBat corrected patient group and for each patient a normative ComBat corrected HC sample within an age window of +/-3 years was found. Then, the median and standard deviation was computed at the feature level and the patients' data were z-standardized. We then trained a repeated nested pooled cross-validation (CV) SVM model with 10 outer CV2 permutations, 10 outer CV2 folds, 10 inner CV1 permutations, and 10 inner CV1 folds using the z-standardized PRONIA data and applied the model to the z-standardized external validation datasets.

1.11 Cluster Significance Testing

Permutation testing is a robust tool for assessing statistical significance of machine learning models[35,36]. We assessed the statistical significance of clusters in three ways. First, we assessed whether the obtained subgroups were different to those which would be found in different disease structures by comparing the observed ARI to a null distribution of ARI created by randomly shuffling features in the HC group and the patient group and permuting 1000 times in HYDRA. Second, we assessed whether the obtained clusters were different to those which would be found if disease-related variability was not present by comparing the observed ARI to a null distribution of ARI created by randomly assigning HC samples (n=275) to a HC group and pseudo-patient group and performing HYDRA analysis 1000 times. Third, we also assessed whether the data could be explained with clusters or if it could be better explained by a single Gaussian distribution by employing SigClust (1000 simulations) (45) (https://github.com/pkimes/sigclust2). SigClust tests the null hypothesis that the data are from a single Gaussian distribution by using a test statistic called the cluster index (ClustI). The ClustI is defined as the sum of within-class sums of squares about the mean divided by the total

sum of squares about the overall mean. The null distribution of the ClustI is approximated by simulating from a single Gaussian distribution estimated from the data.

Due to the unique nature of our recent onset samples we defined the null distribution by disturbing the disease signature in the patient group and the healthy control group. We performed random shuffling of features in the healthy control group and the patient group separately and permuted in HYDRA 1000 times using a repeated hold-out cross-validation strategy (i.e., 10 repetitions with 80% of the data for training in each repetition). We also permuted HYDRA 1000 times using the original data using a repeated hold-out cross-validation strategy (i.e., 10 repetitions with 80% of the data for training in each repetition). Thus, we obtained a null distribution ARI and compared it to the observed ARI. The ARI for the 2 cluster solution was statistically significantly higher in the observed distribution compared to the null distribution (pfdr <.001). ARIs for all other observed clustering solutions showed no statistically significant differences to the null distribution ones (pfdr > 0.001). We also performed permutation testing by randomly assigning HC samples (n=275) to a HC group and pseudo-patient group while maintaining the HC to patient ratio that was observed in the original data and performed HYDRA analysis 1000 times using a repeated hold-out cross-validation strategy (i.e., 10 repetitions with 80% of the data for training in each repetition)[37,38]. We compared the results to clustering results obtained with actual HC and patient groups. The ARI for the 2 cluster solution was statistically significantly higher in the observed distribution compared to the null distribution (pfdr=0.01).

1.12 SVM Details of Independent Validation and Prognostic Validation Models of 9-month GAF-S outcomes.

To further investigate the granularity of the differences between clusters, and manage the challenge of multiple testing in univariate statistics, we also trained Support Vector Machine

(SVM) models to classify patients into the neuroanatomically based clusters using neurocognitive, blood-based biomarker, and symptom data and examined weighted features driving classification within the models. The machine learning analysis of pre-processed data was performed using NeuroMiner (version 1.0; https://github.com/neurominer-git). Prior to the machine learning analysis the following preprocessing steps were completed: a) every feature was scaled from 0 to 1 and completely non-finite features were zeroed-out; b) any feature that had infinite values was pruned; c) missing values were imputed using KNN Euclidean distance median replacement using 7 nearest neighbours. For each missing value of a given CV1 or CV2 subject, a subset of cases that had values for the given variable and had values in all other variables which were non-empty were identified. Subjects in the source subset were sorted according to their similarity with the target subject using the Euclidean distance. Then, the median of the given variable was computed using the 7 nearest neighbours. The original, non-imputed training matrix was used at all times. d) The data was scaled again. The model performance criterion that was used was balanced accuracy and the learning algorithm that was chosen was LIBLINEAR L2-regularized L2-loss SVC. Imbalanced learning was corrected for by increasing the C value in the minority class by multiplying it by the inverse ratio of the training class sizes (weighting the hyperplane). The kernel type that was linear with eleven learning parameters in order to optimize the choice of C value. Wrapper methods were activated at all parameter combinations with greedy sequential backward feature selection (Stop at k=90% of features; Feature stepping at 10% of worst performing features at each cycle). Starting with the full feature set the SVM was ran iteratively with the 10% worst performing features being eliminated at each cycle until 10% of the feature pool was left. In order to assess the models' statistical significance we performed permutation analysis to create an empirical null distribution of weights for each feature and then compare the observed weight to this distribution. The models were retrained in the cross-validation framework using the

respective label subsets obtained from the observed-label analyses 1000 times. For each permutation the predictions were accumulated into a permuted ensemble prediction for each CV2 subject. In that way a null distribution of out-of-training classification performance for the prediction models was produced. The significance of the observed out-of-training classification performance was calculated as the number of events where the permuted out-of-training classification performance was higher or equal to the observed classification performance divided by the number of permutations performed. Then the significance of the model was determined according to a p threshold of $p<0.05$. Furthermore we applied a sign-based consistency algorithm to calculate the number of times that the sign of each feature (positive or negative) was consistent within an ensemble multiplied by the number of times that the feature was non-zero. The measure is between 0 to 1, with 1 representing perfect consistency within the ensemble and 0 if the weights are equally positive and negative or when the feature is omitted with a zero weight. A p-value was then calculated by defining a hypothesis test for the importance score with a null hypothesis of 0. A z-score was calculated as the importance divided by the square root of the variance of the importance scores. A standard p-value was then calculated using a normal cumulative distribution function to choose the right-tailed significance. P-values were then corrected using the false-discovery rate.

A repeated nested pooled cross-validation (CV) was used with 10 outer CV2 permutations, 10 outer CV2 folds, 10 inner CV1 permutations, and 10 inner CV1 folds. Imbalanced learning was corrected for, by increasing the C value in the minority class by multiplying it by the inverse ratio of the training class sizes. A linear kernel was used with 11 C values (0.0156, 0.0312, 0.0625, 0.1250, 0.2500, 0.5000, 1, 2, 4, 8, and 16) to optimize the choice of C value and create an ensemble of predictive models to be applied to the CV2 data to produce a single average robust prediction. Balanced accuracy (BAC) regularized by SVM model complexity

was used as a criterion for the hyperparameter optimization. For hyperparameter optimization, we computed

$\overline{\mathrm{BAC}_{\mathrm{reg}}} = \sum_{i=1}(\frac{n_{TP_i}}{n_{TP_i}+n_{FN_i}} + \frac{n_{TN_i}}{n_{TN_i}+n_{FP_i}})/2$ at given parameter combination across all k CV_1 partitions with $n_{TP_i}/n_{TP_i}+n_{FN_i}$ being Sensitivity and $n_{TN_i}/n_{TN_i}+n_{FP_i}$ being Specificity, and the fraction of the training population serving as support vectors in the ith CV_1 partition. Our optimization technique's aim was to find a combination of T_G, PCs and the SVM's regularization parameter C [range: $2^{[-3 \xrightarrow[\in \mathbb{Z}]{} +4]}$that maximized $\overline{\mathrm{BAC}_{\mathrm{reg}}}$ within a 3 (T_G) × 5 (PC) × 8 (C) hyperparameter cube. The optimized ensemble was then applied to the CV_2 validation data to produce a mean decision score ($\overline{D_{ens}}$) and majority voting-based class membership probabilities (P_{ens}) for each CV_2 validation subject. This produced a mean decision score ((D_ens) ̅) and majority voting-based class membership probabilities (P_ens) for each CV2 validation subject. Finally, a stacking-based data fusion framework[39,40] was used to examine whether the combination of the symptom-based and the blood-based biomarker models would provide a superior classification accuracy. To achieve this, the decision scores of the blood-based biomarker and the symptom-based models were combined, standardized, and forwarded to a greedy sequential forward search algorithm[41] which found a parsimonious combination of classifiers maximizing $\overline{\mathrm{PSI}_{\mathrm{reg}}}$ across the C parameter range by employing L2-regularized logistic regression (L2LR)[42]. Each L2LR ensemble was then applied to the standardized neuroimaging and clinical based decision scores available for the CV_2 validation data. Class prediction was achieved by using majority voting on Majority voting on P_{ens}.

1.13 Prognostic Validation Post-Hoc Results

Post-hoc Mann-Whitney U test results showed statistically significant differences between the BAC of the ROP stacked model and the Preserved Cluster stacked model (U= .00, p<0.001), between the BAC of the ROD stacked model and the Preserved Cluster stacked model (U= .00,

$p<0.001$), and between the Impaired Cluster Stacked model and the Preserved Cluster stacked model (U=.00, $p<0.001$), with no overlap of the values among the respective groups.

1.14 List of Features Included in Prognostic Validation Analysis

The symptom-based support vector machine learning model was trained using item scores the following tests: PANSS, BDI, and GAF-S. The blood-based biomarker model was trained using the following peripheral inflammatory marker data: IL1ra, CRP, TNFα, BDNF, and TGFβ as well as the following additional variables: age, sex, tobacco use, and BMI.

1.15 Multivariate Analyses Results

Using symptom measures, and reflecting univariate results, the two clusters were separable (BAC: 60.9%, sensitivity: 65.4%, specificity: 56.4%, AUC: 0.63, $p<0.01$). Assessing the feature weights of the model, patients in Cluster 2 exhibited higher scores in positive (PANSS-P items 6 and 7; suspiciousness/persecution and hostility), general (PANSS-G items 2 and 13; anxiety and disturbance of volition), negative symptoms (PANSS-N items 1 and 7; blunted affect and stereotyped thinking) and (SANS items 13, 14, 23, 25; alogia, grooming and hygiene, social inattentiveness, and attention) and BDI-II items 16 and 18 (changes in appetite and sleeping patterns) among others. Patients in cluster 1 had higher scores in BDI-II items 4, 5, 8, 14, 15, and 17 (loss of pleasure, guilty feelings, self-criticalness, loss of energy, and irritability) and PANSS-G items 4, 6, 12, 14, and 15 (tension, depression, lack of judgement and insight, and poor impulse control) among others (See figure S3(a)).

Our neuroanatomically based clusters were also separable using cognitive data (BAC: 56.6%, sensitivity: 57.5%, specificity: 55.7%, AUC: 0.58, $p=0.01$). Patients in cluster 2 mainly exhibited worse cognitive performance in a visual recognition and recall task (Rey–Osterrieth complex figure) and patients in cluster 1 mainly performed worse in verbal memory tasks (Rey Auditory Verbal Learning Test) (See supplementary figures S3(b)).

The two clusters were also separable (BAC: 58.7%, sensitivity: 54.9%, specificity: 62.4%, AUC: 0.59, p=0.01) in blood-based biomarkers, with patients in cluster 2 having elevated levels of CRP and TNFα (See supplementary figures S3(c)).

Table S5: Clinical univariate comparisons between the two clusters (fdr-corrected p values)

	Cluster 1 (Preserved)	Cluster 2 (Impaired)	T value	P value
PANSS Positive M (SD)	**11.5 (5.8)**	**13.8 (7.46)**	**-2.8**	**.02**
PANSS Negative M (SD)	**13.5 (6.3)**	**15.2 (13.5)**	**-2.2**	**.04**
PANSS General M (SD)	**29.8 (8.2)**	**33.0 (11.4)**	**-2.7**	**.01**
BDI M (SD)	**25.1 (14.2)**	**22.5 (12.7)**	**1.5**	**.12**
Global Functioning Social Current M (SD)	**6.0 (1.6)**	**5.7 (1.6)**	**1.5**	**.12**
Global Functioning Role Current M (SD)	**5.8 (1.8)**	**5.3 (1.8)**	**-2.3**	**.03**
SANS Affective Flattening M (SD)	**5.3 (7.6)**	**8.1 (9.1)**	**-2.7**	**.01**
SANS Alogia M (SD)	**2.1 (4.1)**	**3.8 (5.3)**	**-3.0**	**.02**
SANS Avolition M (SD)	**5.7 (4.6)**	**6.7 (5.3)**	**-1.7**	**.07**
SANS Anhedonia M (SD)	**8.8 (6.7)**	**9.8 (7.8)**	**-1.1**	**.25**
SANS Attention M (SD)	**1.8 (2.7)**	**2.8 (1.8)**	**-2.2**	**.04**

Table S6. Neurocognitive performance univariate comparisons between the two clusters (fdr-corrected p values)

	Cluster 1 (Preserved)	Cluster 2 (Impaired)	T value	P value
Verbal Fluency M (SD)	22.5 (6.7)	21.9 (7.0)	0.812	0.556
Social Cognition M (SD)	18.9 (2.5)	18.5 (2.7)	1.192	0.430
Working Memory M (SD)	15.6 (4.1)	15.5 (3.7)	0.121	0.904
TMA M (SD)	31.2 (13.3)	35.3 (16.9)	-2.212	0.074
DSST M (SD)	57.2 (12.6)	53.3 (13.9)	2.446	0.068
Reasoning M (SD)	19.6 (4.5)	19 (4.3)	1.107	0.430
Attention M (SD)	254.2 (24.6)	253.4 (20.1)	0.312	0.862
Speed of Processing M (SD)	48.5 (25.8)	40.0 (32.3)	2.399	0.068

Table S7. Blood-based biomarker univariate comparisons between the two clusters (fdr-corrected p values)

	Impaired Cluster	Preserved Cluster	T value	P value
IFN-γ M (SD)	4.2 (13.8)	2.8 (3.9)	-1.049	.296
IL1-ra M (SD)	615.4 (541.2)	802.3 (1304.6)	1.257	.210
IL-4 M (SD)	10.5 (10.8)	9.5 (8.6)	-.686	.493
S100B M (SD)	44.4 (61.1)	48.7 (82.4)	.409	.683
IL1-β M (SD)	.92 (.82)	3.0 (23.5)	.862	.390
IL-2 M (SD)	1.4 (1.9)	1.2 (1.7)	-.851	.396
IL-6 M (SD)	.83 (1.6)	.81 (.83)	-.072	.942
TNF-α M (SD)	1.6 (1.2)	1.6 (1.2)	.022	.983
CRP M (SD)	1562227.8 (4294394.8)	1380328.4 (1892965.3)	-.394	.694
TGF-β M (SD)	375230.8 (384248.7)	369705.3 (369923.8)	-.102	.919
BDNF M (SD)	21604.4 (9481.6)	22859.5 (9364.3)	.926	.356

Table S8. Results of VBM comparison between clusters and HC

Comparison	Cluster Peak Voxel MNI Coordinates (x,y,z)	P Value, Peak Intensity, Cluster Size
HC < Cluster 1		
Bilateral Cerebellum, Left Lingual Gyrus, Fusiform Gyrus	-8 -54 -18	p(FDR)<0.001, T=4.01, k=1164
Bilateral Cerebellum	-2 -63 -30	p(FDR)=0.011, T=4.05, k=491
HC > Cluster 2		
Superior Temporal Gyrus, Right Insula, Right Superior Temporal Gyrus, Middle Temporal Gyrus, Parahippocampal Gyrus, Right Hippocampus, Inferior Frontal Gyrus, Right Amygdala, Fusiform Gyrus	53 6 -3	p(FDR)<0.001, T=5.46, k=9134
Anterior Cingulate, Medial Frontal Gyrus, Left Rectal Gyrus	-9 42 -3	p(FDR)<0.001, T=5.03, k=10983
Precuneus, Middle Temporal Gyrus,	-26 -68 27	p(FDR)<0.001, T=4.78, k=1376
Left Superior Temporal Gyrus, Left Insula, Left Inferior Frontal Gyrus, Left Hippocampus, Left Claustrum	-36 -9 14	p(FDR)<0.001, T=4.74, k=7684
Bilateral Precuneus, Cingulate Gyrus, Posterior Cingulate	11 -51 29	p(FDR)<0.001, T=4.54, k=1390
Superior Frontal Gyrus, Medial Frontal Gyrus	17 57 9	p(FDR)<0.001, T=4.36, k=1605
Bilateral Thalamus	0 -12 -5	p(FDR)=0.009, T=4.17, k=405
Right Postcentral Gyrus, Right Precentral Gyrus	26 -41 47	p(FDR)=0.010, T=4.12, k=386

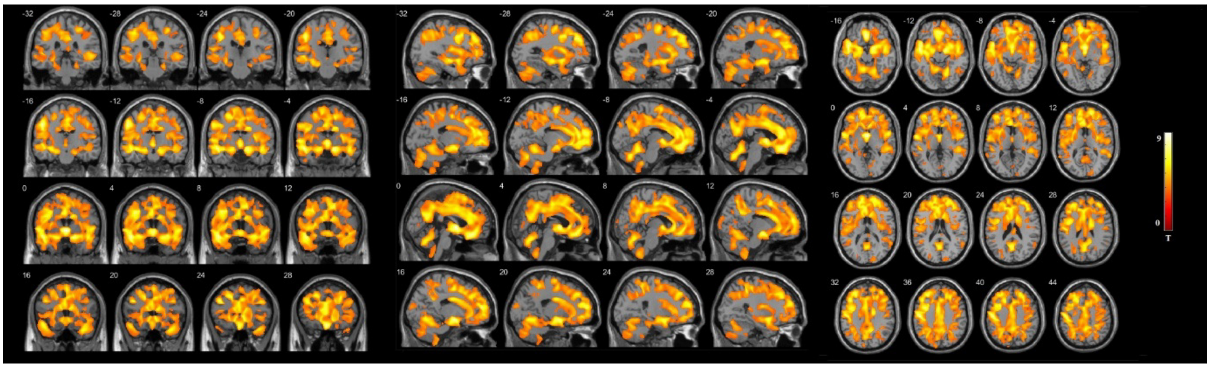

(a)

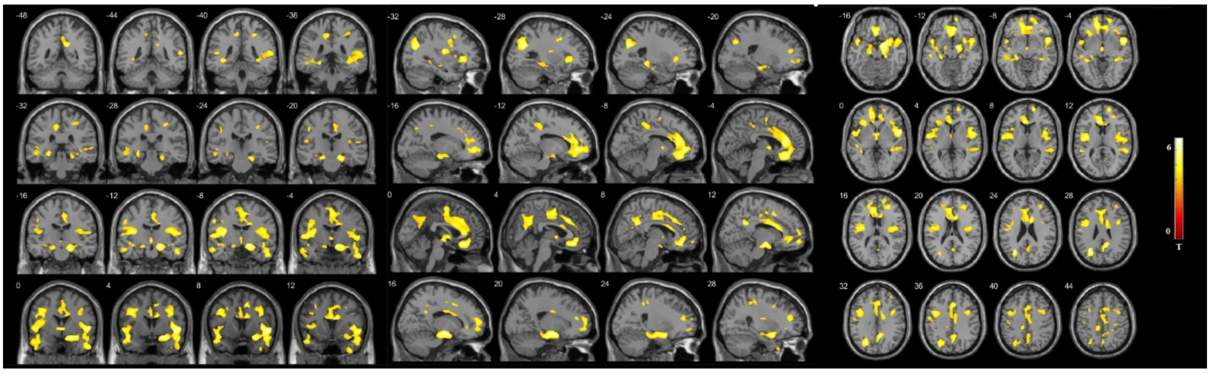

(b)

Figure S2. GMV decreases between Impaired cluster and Preserved cluster (a) and between Impaired cluster and HC (b).

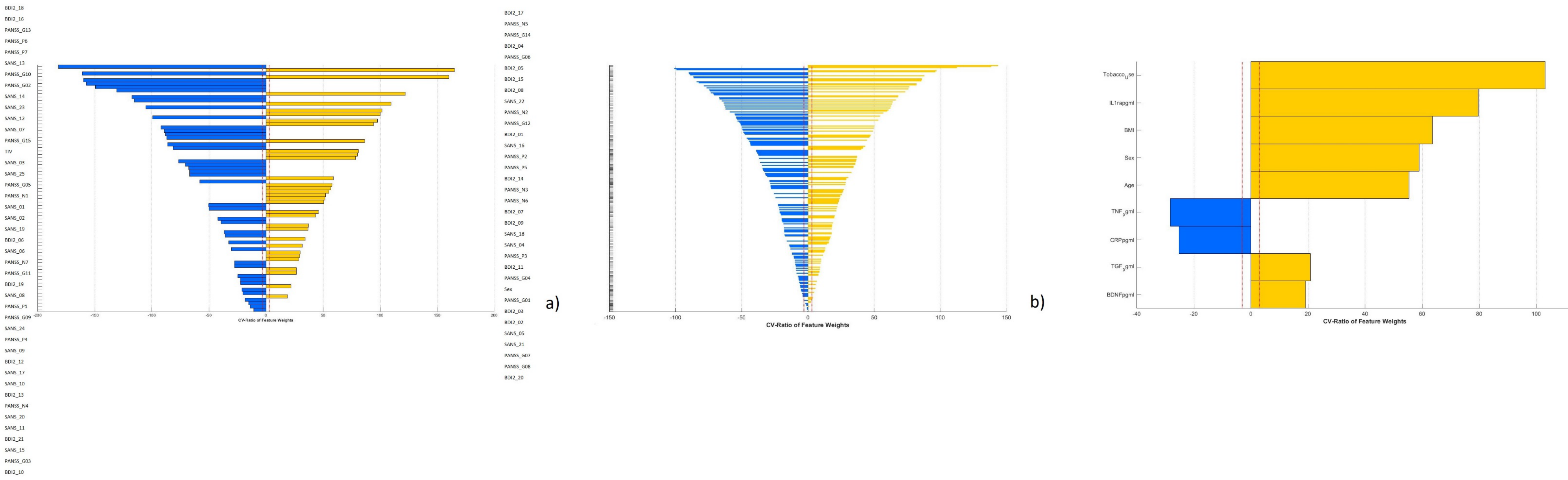

Figure S3. CV ratio of Feature Weights of SVM Classification between the Two Clusters Using Symptom Measures (a), cognitive variables (b)*, and blood-based biomarker data (c). Features in blue denote importance in impaired cluster classification and features in yellow denote importance in preserved cluster classification.

*The cognitive variables included in the model were (from top to bottom/most important to least important):

GAVLT_Delayed_repetition_out_of_list_words_list_A_T0

GROCF_Score_element_09_T0

GROCF_Score_element_05_Delayed_T0

GPVF_Correct_00_15_letter_1_T0

GFDS_Maxdigitsstringlengthcorrect_reminded_at_least_o_T0

GDSST_Score_symbol_matchings_T0

GDSST_Correct_number_symbol_matchings_T0

GROCF_Score_element_13_T0

GAVLT_Immedirepeatedwords1repetitionlistA_T0

GSVF_Correct_45_60_category_1_T0

SOPT_Maxcorrectresponsesbeforeerr10elem02_T0

GPVF_Repetition_15_30_letter_1_T0

GSVF_Error_45_60_category_1_T0

GROCF_Score_element_07_Immediate_T0

CPTIP_Number_error_filler_stimuli_200_trials_T0

SOPT_Perseveration_Errors_10_elements_03_T0

GROCF_Score_element_18_Delayed_T0

GROCF_Time_T0

CPTIP_Number_error_distracting_stimuli_50_trials_T0

Age

GAVLT_Immedioutoflist_words1repetitionlistA_T0

GPVF_Repetition_00_15_letter_1_T0

CPTIP_Reaction_times_error_distracting_300_trials_T0

GAVLT_Immedioutoflistwords5repetitionlistA_T0

GTMB_2_Errors_T0

GROCF_Score_element_06_T0

GSVF_Correct_30_45_category_1_T0

SOPT_Errors_10_elements_02_T0

GSVF_Repetition_30_45_category_1_T0

SOPT_Perseveration_Errors_6_elements_03_T0

GROCF_Score_element_16_Immediate_T0

GAVLT_Interference_Immediate_6_repetition_list_A_T0

GSVF_Repetition_00_60_category_1_T0

CPTIP_Number_correct_250_trials_T0

GAVLT_Immediate_5_repetition_list_A_T0

SOPT_Maxcorrectresponsesbeforeerr6elem01_T0

GAVLT_Immedirepeatedwords3repetitionlistA_T0

GTMA_1_Time_of_execution_T0

GROCF_Score_element_02_T0

GSVF_Repetition_15_30_category_1_T0

GROCF_Score_element_15_Immediate_T0

SOPT_Maxcorrectresponsesbeforeerr8elem01_T0

CPTIP_Number_error_filler_stimuli_100_trials_T0

GAVLT_Immedirepeatedwords2repetitionlistA_T0

GPVF_Error_45_60_letter_1_T0

CPTIP_Number_error_filler_stimuli_250_trials_T0

GROCF_Score_element_10_Immediate_T0

CPTIP_Reaction_times_error_distracting_100_trials_T0

GSVF_Correct_15_30_category_1_T0

GAVLT_Immedirepeatedwords4repetitionlistA_T0

GROCF_Score_element_14_Immediate_T0

GSAT_objects_rt_mean_T0

SOPT_Maxcorrectresponsesbeforeerr8elem03_T0

GROCF_Score_element_17_Immediate_T0

CPTIP_Number_error_distracting_stimuli_250_trials_T0

GPVF_Repetition_45_60_letter_1_T0

GROCF_Score_element_06_Delayed_T0

GTMA_3_Violations_T0

CPTIP_Number_error_distracting_stimuli_100_trials_T0

GSAT_animals_rt_sd_T0

GROCF_Score_element_02_Delayed_T0

GSAT_blue_objects_rt_mean_T0

GAVLT_Immediate_2_repetition_list_A_T0

GROCF_Score_element_14_T0

GROCF_Score_element_10_T0

CPTIP_Number_error_filler_stimuli_whole_test_T0

GROCF_Time_Delayed_T0

CPTIP_Reaction_times_error_distracting_200_trials_T0

CPTIP_Number_correct_200_trials_T0

GPVF_Correct_45_60_letter_1_T0

SOPT_Maxcorrectresponsesbeforeerr4elem02_T0

GROCF_Score_element_12_Delayed_T0

SOPT_Perseveration_Errors_4_elements_01_T0

GTMB_1_Time_of_execution_T0

CPTIP_Reaction_times_error_distracting_250_trials_T0

GROCF_Score_element_13_Delayed_T0

GFDS_Number_of_correct_trials_T0

SOPT_Maxcorrectresponsesbeforeerr10elem01_T0

GROCF_Placement_whole_Delayed_T0

GROCF_Score_element_04_T0

GROCF_Score_element_03_Delayed_T0

CPTIP_Reaction_times_error_distracting_150_trials_T0

GSAT_red_animals_vas_T0

CPTIP_Number_error_filler_stimuli_150_trials_T0

GTMB_3_Violations_T0

GROCF_Score_element_18_T0

GROCF_Score_element_08_Delayed_T0

WAIS_V_Standard_score_T0

GROCF_Score_element_11_Immediate_T0

WAIS_V_Raw_score_T0

SOPT_Errors_8_elements_02_T0

GROCF_Score_element_11_Delayed_T0

GSAT_red_objects_rt_mean_T0

GAVLT_InterfeImmedioutoflistwords6rep_lis_T0

CPTIP_Reaction_times_correct_50_trials_T0

GSVF_Error_00_15_category_1_T0

GROCF_Score_element_11_T0

GSAT_red_objects_rt_sd_T0

SOPT_Maxcorrectresponsesbeforeerr10elem03_T0

SOPT_Perseveration_Errors_8_elements_01_T0

SOPT_Maxcorrectresponsesbeforeerr6elem03_T0

GPVF_Error_00_60_letter_1_T0

GPVF_Error_15_30_letter_1_T0

GSVF_Error_00_60_category_1_T0

GSVF_Error_30_45_category_1_T0

GBDS_Maxdigitsstringlengthcorrect_reminded_at_least_o_T0

WAIS_MR_Standard_score_T0

SOPT_Perseveration_Errors_4_elements_03_T0

GROCF_Score_element_16_T0

SOPT_Maxcorrectresponsesbeforeerr8elem02_T0

GSAT_red_animals_rt_sd_T0

GSAT_blue_objects_rt_sd_T0

SOPT_Errors_10_elements_01_T0

GAVLT_Immedirepeatedwords5repetitionlistA_T0

GROCF_Score_element_07_T0

SOPT_Errors_6_elements_02_T0

Sex

GSAT_red_rt_mean_T0

CPTIP_Number_omissions_250_trials_T0

GROCF_Accuracy_whole_T0

GAVLT_InterfeImmediwordsfromlistArepetitionlist_T0

SOPT_Errors_6_elements_03_T0

GROCF_Score_element_04_Delayed_T0

GROCF_Score_element_17_T0

SOPT_Maxcorrectresponsesbeforeerr4elem03_T0

GAVLT_Immediate_4_repetition_list_A_T0

GROCF_Score_element_14_Delayed_T0

CPTIP_Number_omissions_300_trials_T0

GAVLT_InterfeImmedirepeatwordsrepetitionlistB_T0

GROCF_Score_element_15_T0

GROCF_Placement_whole_T0

GROCF_Score_element_08_Immediate_T0

GPVF_Correct_30_45_letter_1_T0

SOPT_Perseveration_Errors_6_elements_02_T0

CPTIP_Number_correct_300_trials_T0

GROCF_Score_element_03_Immediate_T0

SOPT_Errors_10_elements_03_T0

GPVF_Error_00_15_letter_1_T0

GAVLT_InterfeImmediwordsfromlistB6repetitionlis_T0

GAVLT_Immedioutoflistwords2repetitionlistA_T0

GROCF_Score_element_12_T0

CPTIP_Number_error_distracting_stimuli_150_trials_T0

GSAT_objects_rt_sd_T0

GROCF_Score_element_05_T0

GAVLT_Delayed_repetition_list_A_T0

GSAT_red_rt_sd_T0

GAVLT_InterfeImmedirepeatwords6repetitionlistA_T0

GROCF_Accuracy_whole_Immediate_T0

CPTIP_Number_omissions_50_trials_T0

GAVLT_InterfeImmedioutoflistwordsrepetitionlist_T0

GROCF_Score_element_07_Delayed_T0

SOPT_Errors_4_elements_01_T0

GPVF_Repetition_00_60_letter_1_T0

GPVF_Correct_00_60_letter_1_T0

GROCF_Score_element_10_Delayed_T0

GSAT_blue_objects_vas_T0

GROCF_Score_element_09_Delayed_T0

CPTIP_Number_correct_100_trials_T0

CPTIP_Reaction_times_correct_250_trials_T0

SOPT_Errors_8_elements_03_T0

GROCF_Score_element_09_Immediate_T0

SOPT_Errors_4_elements_03_T0

GPVF_Correct_15_30_letter_1_T0

CPTIP_Number_correct_whole_test_T0

GROCF_Score_whole_T0

CPTIP_Reaction_times_error_distracting_whole_test_T0

CPTIP_Reaction_times_correct_300_trials_T0

GSVF_Correct_00_15_category_1_T0

CPTIP_Number_omissions_150_trials_T0

GSAT_blue_rt_mean_T0

GPVF_Error_30_45_letter_1_T0

GSAT_blue_animals_rt_mean_T0

SOPT_Errors_4_elements_02_T0

SOPT_Errors_6_elements_01_T0

CPTIP_Number_error_distracting_stimuli_300_trials_T0

GSAT_animals_rt_mean_T0

GROCF_Score_element_08_T0

CPTIP_Reaction_times_correct_whole_test_T0

GROCF_Placement_whole_Immediate_T0

GROCF_Score_element_17_Delayed_T0

GROCF_Score_element_01_T0

SOPT_Maxcorrectresponsesbeforeerr6elem02_T0

CPTIP_Number_error_filler_stimuli_300_trials_T0

GAVLT_Delayed_repetition_words_from_list_B_list_A_T0

CPTIP_Number_omissions_200_trials_T0

SOPT_Maxcorrectresponsesbeforeerr4elem01_T0

GROCF_Score_element_05_Immediate_T0

GROCF_Score_element_01_Delayed_T0

GSVF_Error_15_30_category_1_T0

CPTIP_Reaction_times_correct_100_trials_T0

GROCF_Score_element_06_Immediate_T0

GSAT_total_rt_mean_T0

GROCF_Score_whole_Immediate_T0

GSAT_blue_animals_vas_T0

CPTIP_Number_error_distracting_stimuli_whole_test_T0

WAIS_MR_Raw_score_T0

CPTIP_Number_correct_150_trials_T0

GROCF_Score_element_13_Immediate_T0

GAVLT_Immedioutoflistwords4repetitionlistA_T0

GSVF_Correct_00_60_category_1_T0

CPTIP_Number_error_distracting_stimuli_200_trials_T0

SOPT_Perseveration_Errors_8_elements_02_T0

SOPT_Perseveration_Errors_8_elements_03_T0

CPTIP_Number_correct_50_trials_T0

GAVLT_Interference_Immediate_repetition_list_B_T0

SOPT_Perseveration_Errors_4_elements_02_T0

GROCF_Score_element_18_Immediate_T0

GSVF_Repetition_00_15_category_1_T0

GROCF_Score_element_12_Immediate_T0

GAVLT_Immediate_3_repetition_list_A_T0

GROCF_Score_element_15_Delayed_T0

CPTIP_Reaction_times_correct_200_trials_T0

CPTIP_Reaction_times_correct_150_trials_T0

CPTIP_Reaction_times_error_filler_whole_test_T0

CPTIP_Number_error_filler_stimuli_50_trials_T0

GROCF_Score_element_03_T0

GAVLT_Delayed_repetition_repeated_words_list_A_T0

GBDS_Number_of_correct_trials_T0

SOPT_Perseveration_Errors_6_elements_01_T0

GSAT_blue_animals_rt_sd_T0

GROCF_Score_element_04_Immediate_T0

SOPT_Perseveration_Errors_10_elements_01_T0

GSAT_blue_rt_sd_T0

GROCF_Accuracy_whole_Delayed_T0

GSAT_red_objects_vas_T0

GROCF_Time_Immediate_T0

GPVF_Repetition_30_45_letter_1_T0

GROCF_Score_element_16_Delayed_T0

GSAT_red_animals_rt_mean_T0

GDSST_Error_number_symbol_matchings_T0

GAVLT_Recognition_words_from_list_A_T0

CPTIP_Number_omissions_100_trials_T0

CPTIP_Number_omissions_whole_test_T0

GAVLT_Immedioutoflistwords3repetitionlistA_T0

GTMA_2_Errors_T0

GSAT_total_rt_sd_T0

SOPT_Perseveration_Errors_10_elements_02_T0

GAVLT_Immediate_1_repetition_list_A_T0

SOPT_Errors_8_elements_01_T0

CPTIP_Reaction_times_error_distracting_50_trials_T0

GDANVA_Number_correct_Faces_condition_T0

GROCF_Score_element_01_Immediate_T0

GROCF_Score_element_02_Immediate_T0

GSVF_Repetition_45_60_category_1_T0

GROCF_Score_whole_Delayed_T0

1.16 Error bar graphs of univariate analyses Discovery data

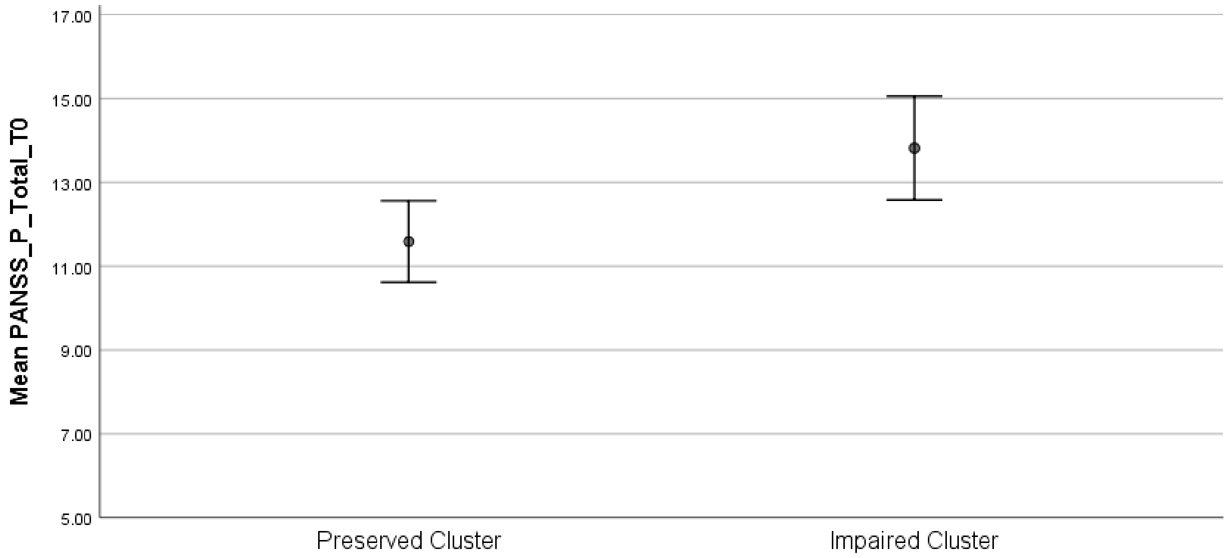

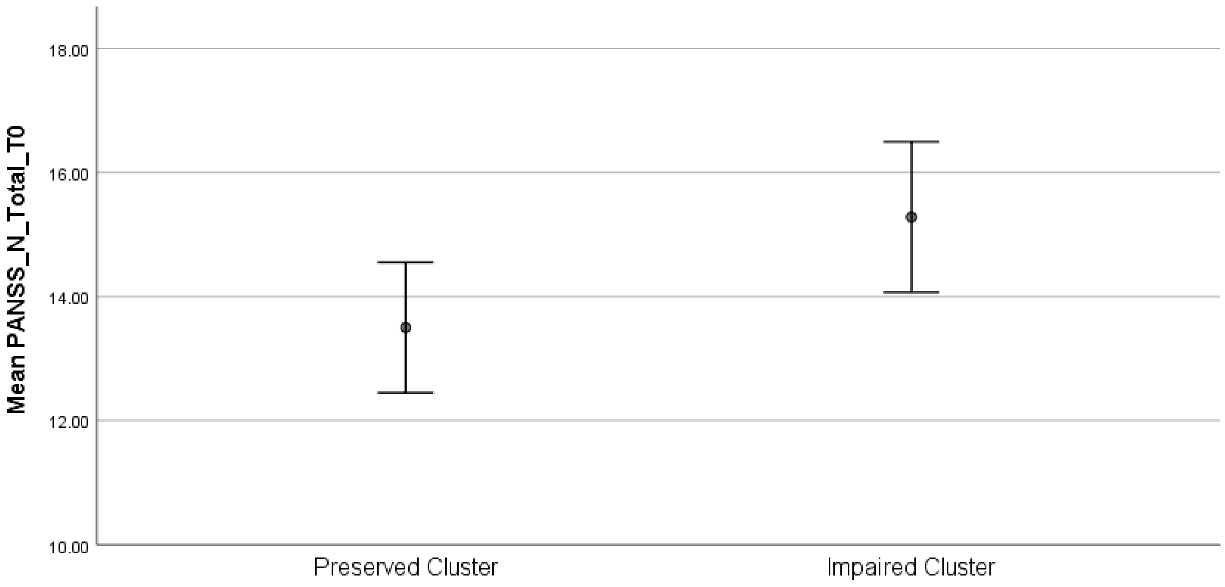
Mean PANSS_N_Total_T0
18.00
16.00
14.00
12.00
10.00
Preserved Cluster
Impaired Cluster

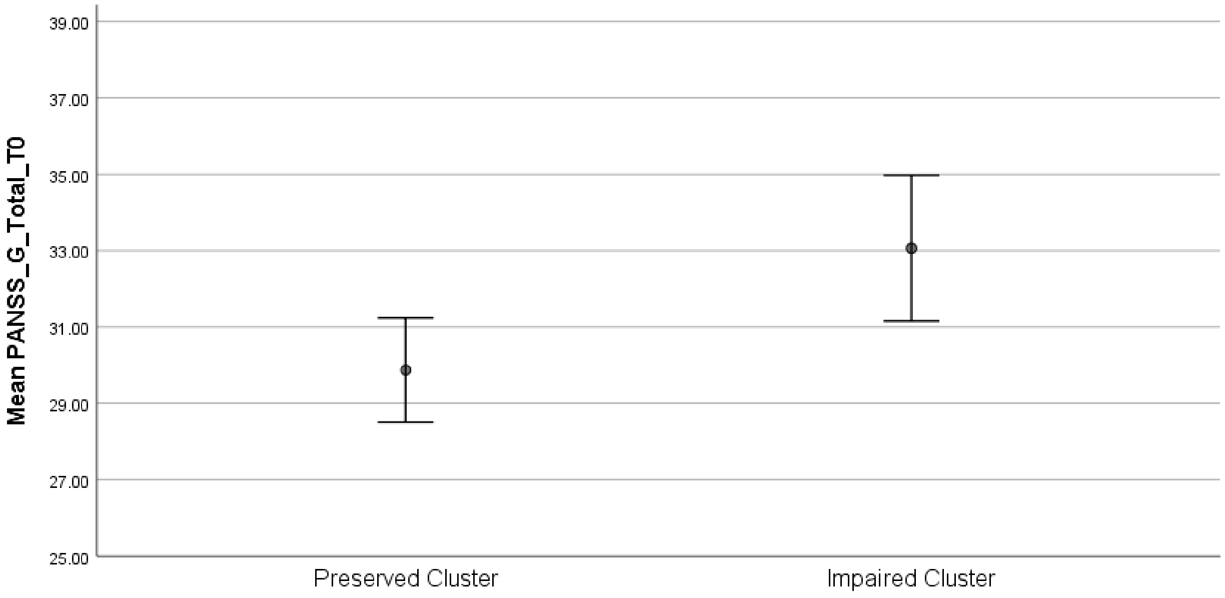
Mean PANSS_G_Total_T0
39.00
37.00
35.00
33.00
31.00
29.00
27.00
25.00
Preserved Cluster
Impaired Cluster

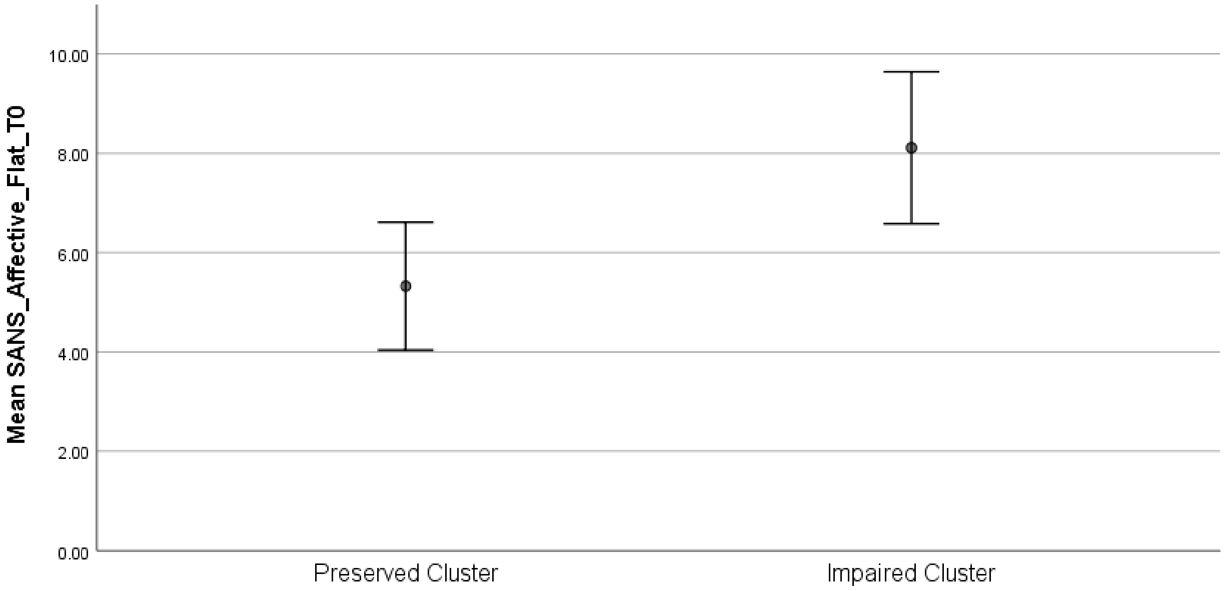
Mean SANS_Affective_Flat_T0
10.00
8.00
6.00
4.00
2.00
0.00
Preserved Cluster
Impaired Cluster

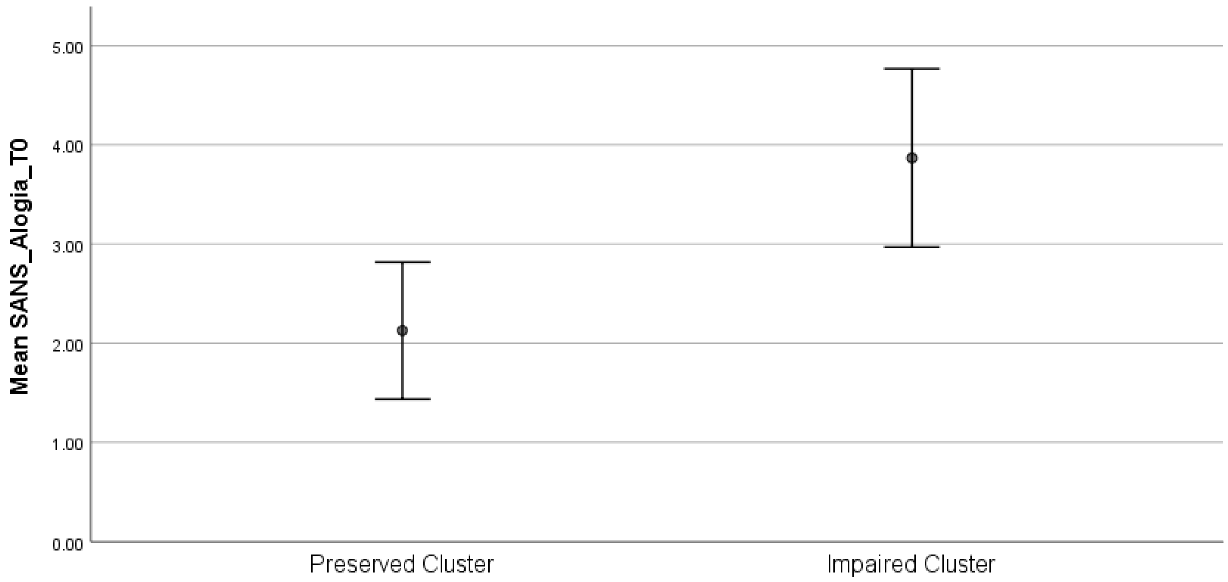
Mean SANS_Alogia_T0
5.00
4.00
3.00
2.00
1.00
0.00
Preserved Cluster
Impaired Cluster

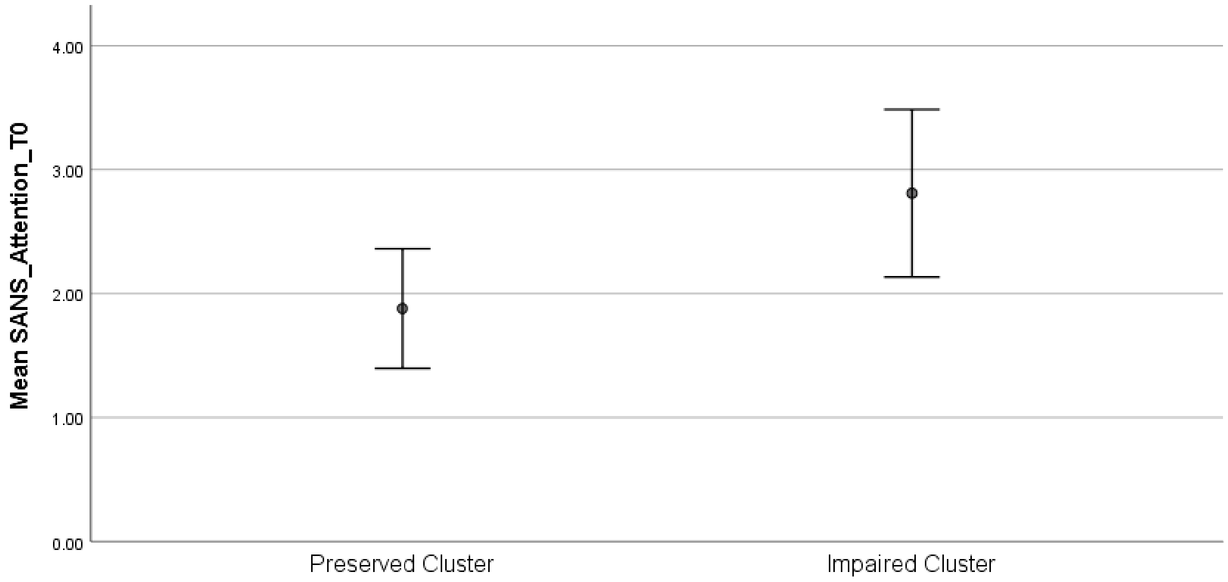
Mean SANS_Attention_T0
4.00
3.00
2.00
1.00
0.00
Preserved Cluster
Impaired Cluster

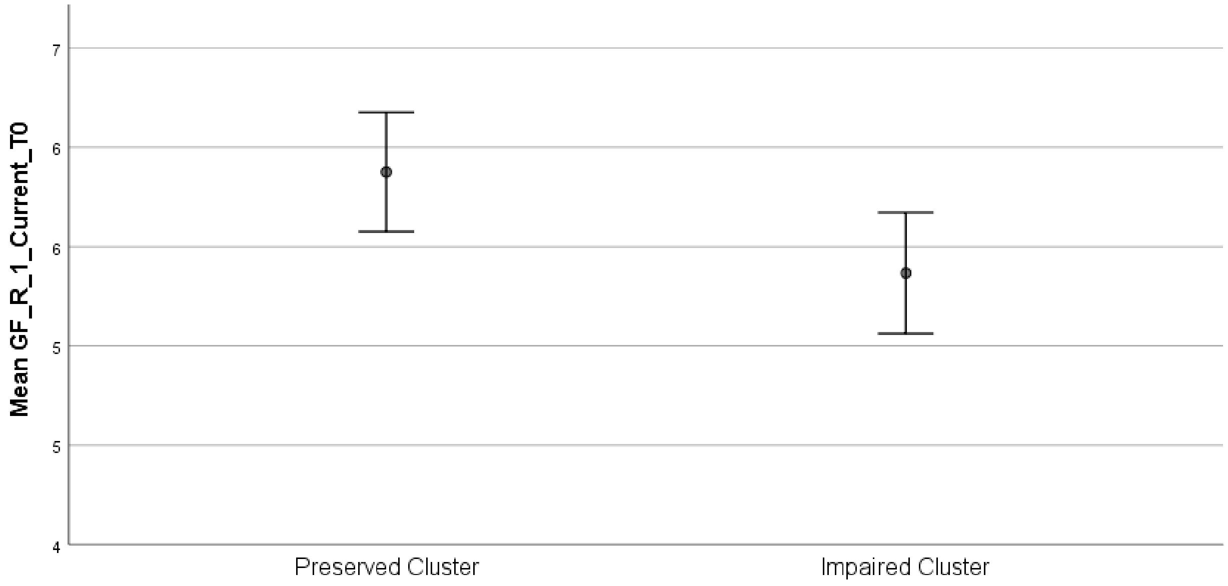
Mean GF_R_1_Current_T0
7
6
6
5
5
4
Preserved Cluster
Impaired Cluster

1.17 ARI significance testing error bars

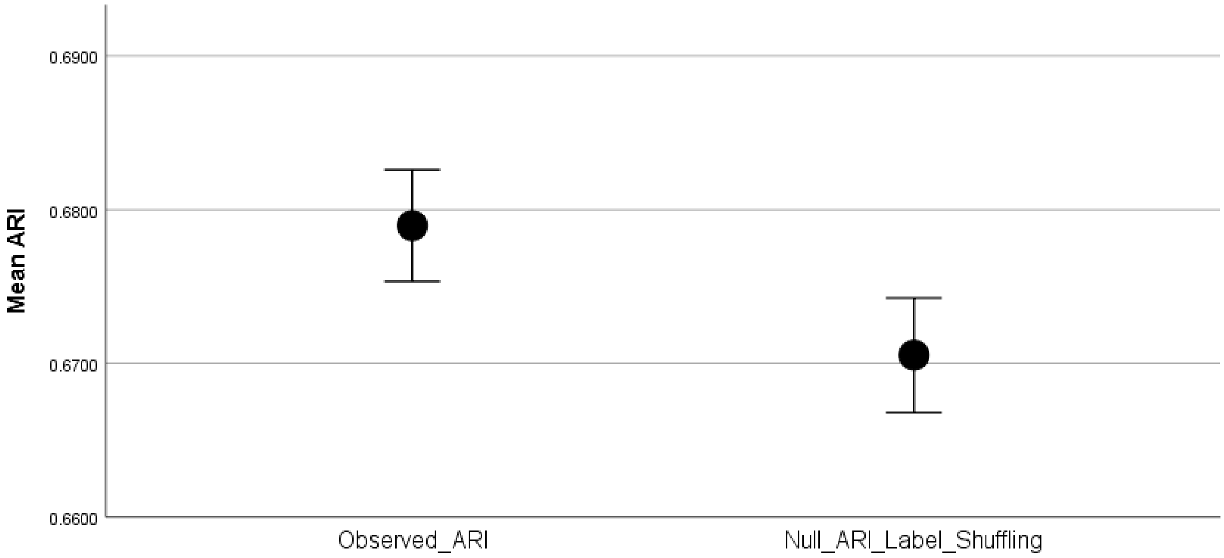

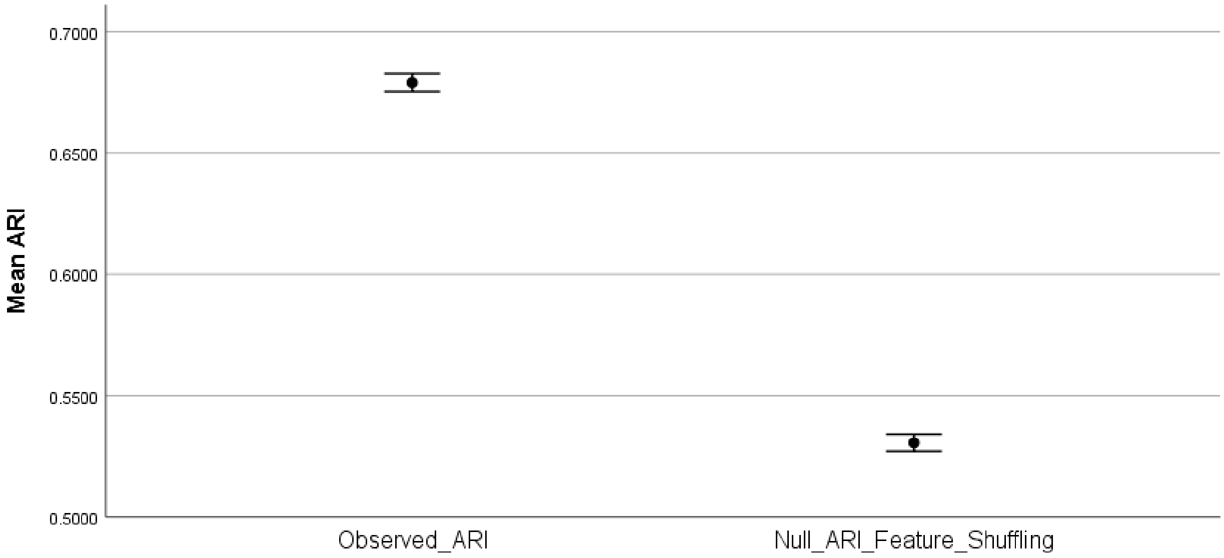

1.18 Independent Validation VBM and Univariate analyses

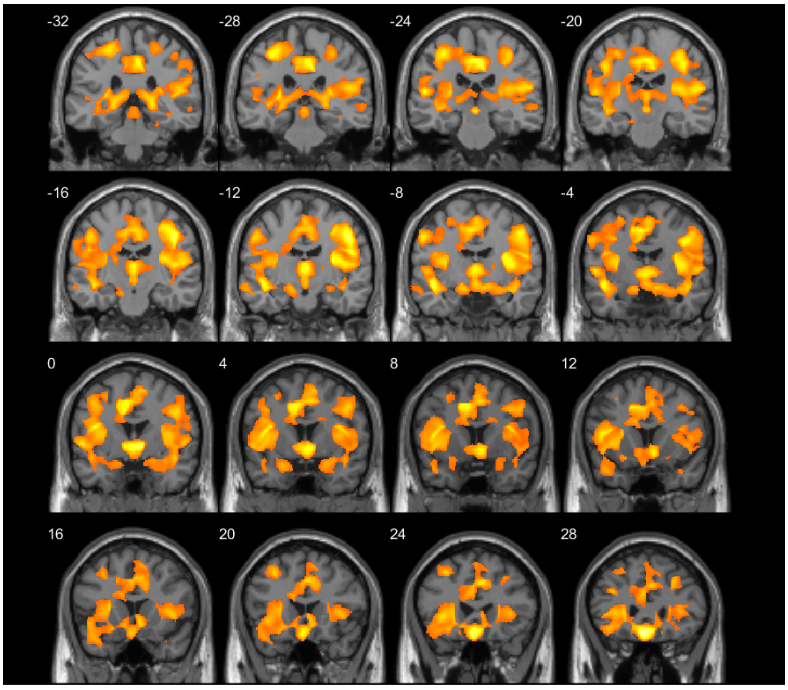

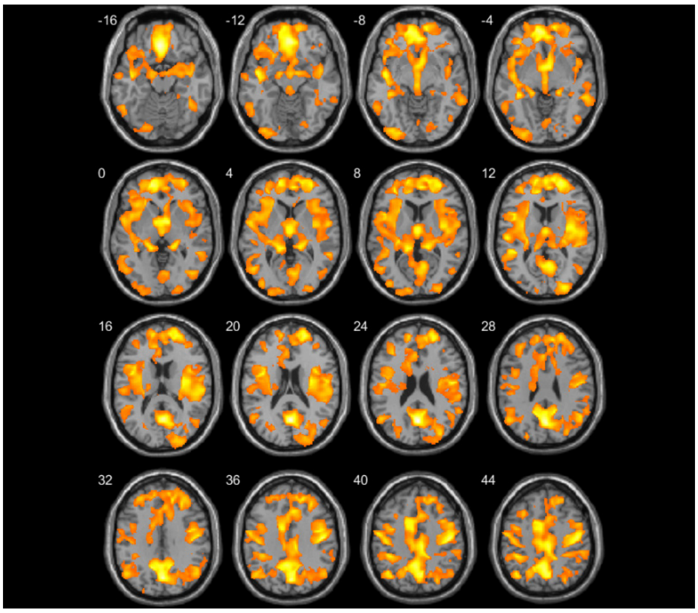

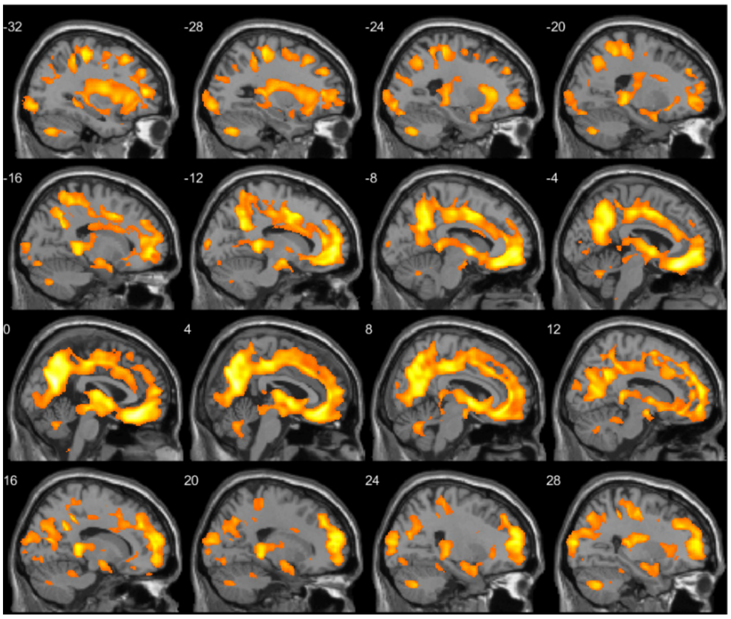

cluster-level			peak-level					mm mm mm		
$q_{FDR-corr}$	k_E	p_{uncorr}	$p_{FWE-corr}$	$q_{FDR-corr}$	T	(Z_E)	p_{uncorr}			
**0.000**	**125211**	**0.000**	**0.000**	**0.000**	**8.11**	**7.57**	**0.000**	**2**	**-65**	**23**
			0.000	0.000	7.56	7.12	0.000	-2	32	-17
			0.000	0.000	7.34	6.93	0.000	3	-72	35
**0.000**	**2949**	**0.000**	**0.014**	**0.003**	**5.39**	**5.22**	**0.000**	**-35**	**-89**	**-9**
			0.056	0.008	5.07	4.92	0.000	-26	-92	-9
			0.376	0.032	4.53	4.42	0.000	-39	-87	6
**0.000**	**1434**	**0.000**	**0.063**	**0.008**	**5.04**	**4.89**	**0.000**	**54**	**-45**	**2**
			0.102	0.011	4.91	4.78	0.000	59	-41	-5
			0.987	0.174	3.87	3.80	0.000	63	-26	-3
**0.000**	**1850**	**0.000**	**0.075**	**0.009**	**4.99**	**4.85**	**0.000**	**29**	**-66**	**-38**
			0.482	0.042	4.44	4.34	0.000	8	-63	-30
			0.663	0.060	4.29	4.20	0.000	-2	-62	-29
**0.000**	**1362**	**0.000**	**0.094**	**0.010**	**4.93**	**4.80**	**0.000**	**-27**	**-68**	**-38**
			1.000	0.392	3.54	3.49	0.000	-42	-56	-45
			1.000	0.772	3.25	3.21	0.001	-32	-54	-39
**0.000**	**830**	**0.000**	**0.260**	**0.024**	**4.65**	**4.53**	**0.000**	**42**	**-50**	**-18**
			0.977	0.155	3.92	3.85	0.000	39	-33	-12
			1.000	0.294	3.66	3.60	0.000	38	-38	-5
**0.004**	**290**	**0.002**	**0.718**	**0.066**	**4.25**	**4.16**	**0.000**	**-62**	**-12**	**-14**
**0.004**	**307**	**0.001**	**0.981**	**0.161**	**3.90**	**3.83**	**0.000**	**20**	**-78**	**-11**
**0.003**	**326**	**0.001**	**0.996**	**0.205**	**3.80**	**3.73**	**0.000**	**14**	**-44**	**-23**

Impaired<Preserved VBM Comparison

Cluster Peak Voxel MNI Coordinates (x,y,z)	
2 -65 23	Medial Frontal Gyrus, Precuneus, Cingulate Gyrus, Precentral Gyrus, Insula, Superior Frontal Gyrus, Middle

	Frontal Gyrus, Superior Temporal Gyrus, Inferior Frontal Gyrus, Left Precuneus, Postcentral Gyrus, Right Precuneus, Anterior Cingulate, Thalamus, Parahippocampal Gyrus
-35 -89 -9	Middle Occipital Gyrus, Inferior Occipital Gyrus, Left Lingual Gyrus
54 -45 2	Middle Temporal Gyrus, Superior Temporal Gyrus
29 -66 -38	Right Cerebellum
-27 -68 -38	Left Cerebellum
42 -50 -18	Fusiform Gyrus
-62 -12 -14	Middle Temporal Gyrus
20 -78 -11	Right Lingual Gyrus
14 -44 -23	Right Cerebellum Anterior Lobe

		t-test for Equality of Means						
							95% Confidence Interval of the Difference	
		t	df	Sig. (2-tailed)	Mean Difference	Std. Error Difference	Lower	Upper
GF_S_1_Current_T0		-1.179	222	.240	-.263	.223	-.701	.176
GF_R_1_Current_T0		-.497	222	.620	-.138	.279	-.688	.411
IFN-γ (pg/ml)		.136	141	.892	.14891	1.09096	-2.00784	2.30566
IL-1ra (pg/ml)		.711	141	.478	57.61485	80.99184	-102.50047	217.73016
IL-4 (pg/ml)		-.528	141	.599	-.40099	.76014	-1.90373	1.10175
S100B (pg/ml)		.432	141	.667	4.82937	11.18814	-17.28882	26.94756
IL-1β (pg/ml)		.702	141	.484	.14544	.20713	-.26405	.55493

IL-2 (pg/ml)		.369	141	.713	.14793	.40105	-.64491	.94078
IL-6 (pg/ml)		1.025	141	.307	.15718	.15336	-.14601	.46037
TNF-α (pg/ml)		-.221	141	.826	-.05614	.25430	-.55888	.44660
CRP (pg/ml)		1.707	141	.090	523068.11532	306451.25281	-82765.01609	1128901.24672
TGF -β (pg/ml)		1.013	141	.313	8729.78934	8621.03783	-8313.41182	25772.99049
BDNF (pg/ml)		.298	141	.766	281.49427	944.93808	-1586.58357	2149.57211
PANSS_P_T0_Total		2.452	213	.015**	2.51082	1.02398	.49240	4.52924
PANSS_N_T0_Total		1.586	212	.114	1.54603	.97489	-.37568	3.46774
PANSS_G_T0_Total		2.114	212	.036	2.95492	1.39794	.19928	5.71056
SANS_Affective_Flattening_T0		3.017	215	.003*	3.98277	1.31990	1.38117	6.58436
SANS_Alogia_T0		3.066	215	.002*	2.27507	.74191	.81271	3.73743
SANS_Avolition_T0		.510	215	.611	.39708	.77870	-1.13777	1.93194

SANS_Anhedonia_T0		.259	210	.796	.29330	1.13028	-1.93486	2.52146
SANS_Attention_T0		3.415	197	.001*	1.72847	.50614	.73031	2.72663

*False Discovery Rate corrected p values: 0.021

**False Discovery Rate corrected p value: 0.078

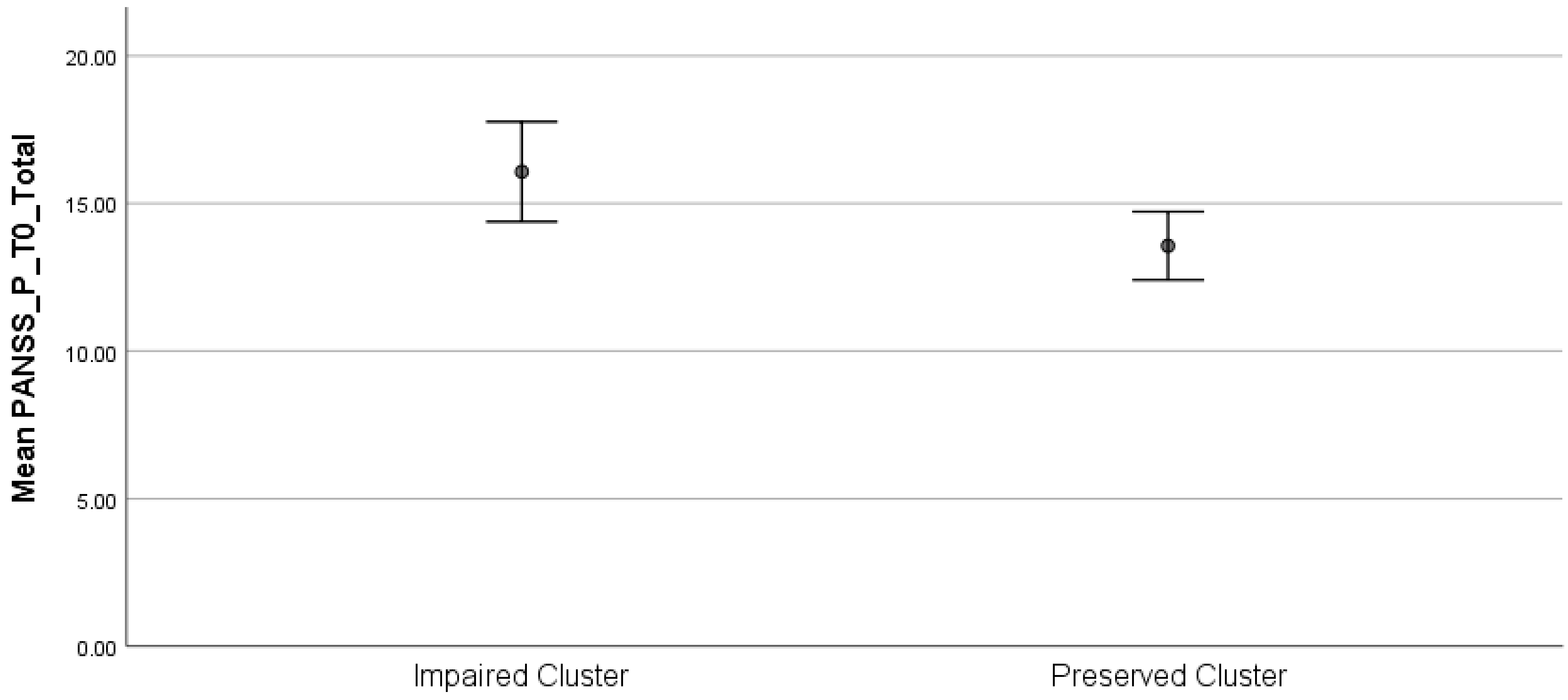
Mean PANSS_P_T0_Total
20.00
15.00
10.00
5.00
0.00
Impaired Cluster
Preserved Cluster

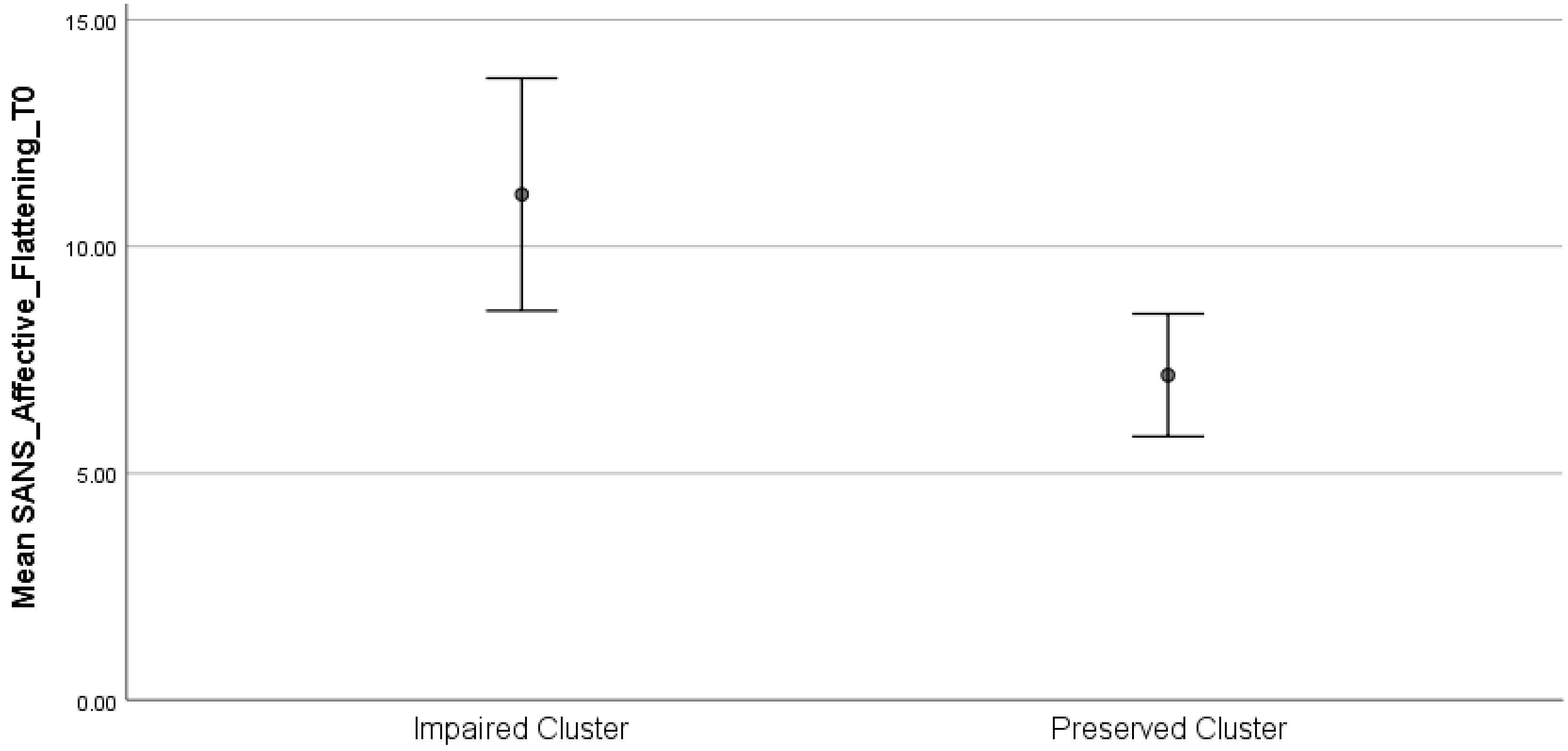
Mean SANS_Affective_Flattening_T0
15.00
10.00
5.00
0.00
Impaired Cluster
Preserved Cluster

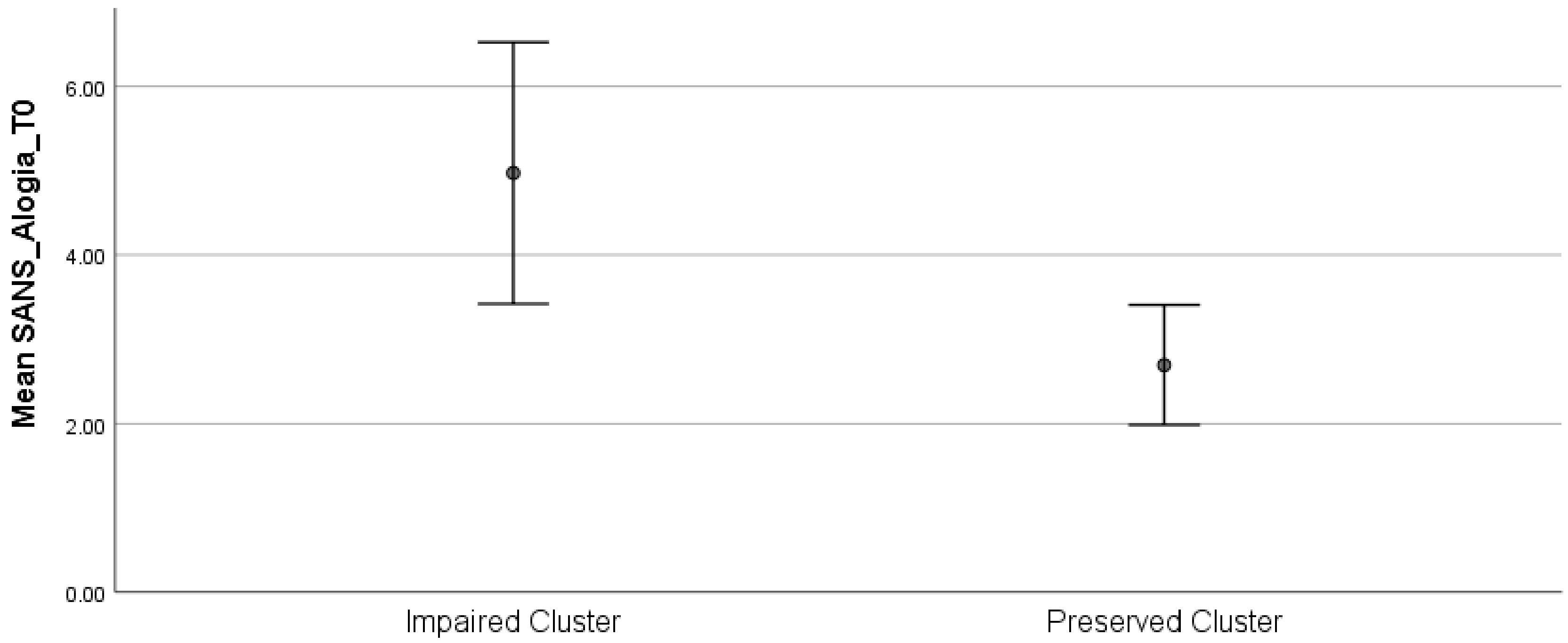
Mean SANS_Alogia_T0
6.00
4.00
2.00
0.00
Impaired Cluster
Preserved Cluster

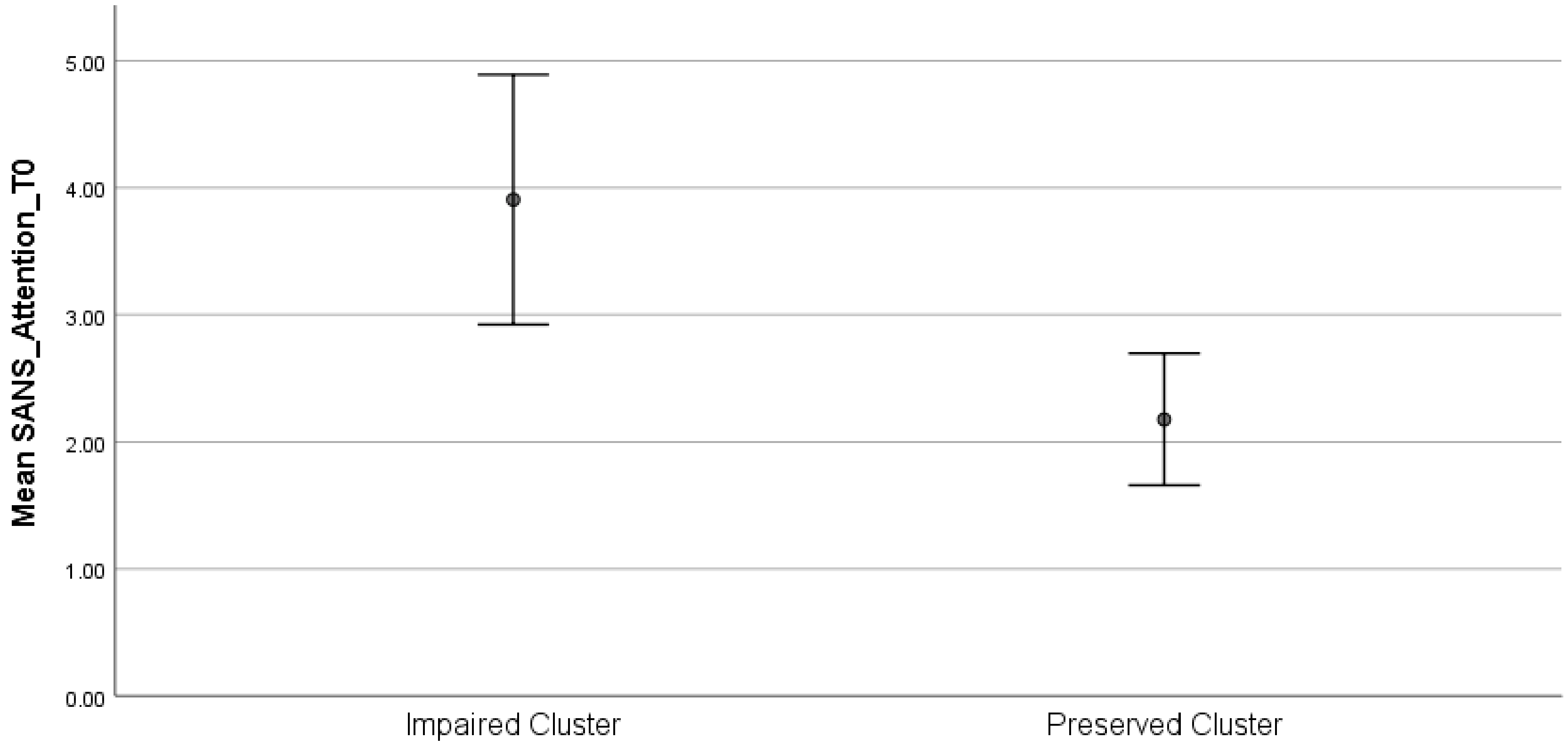
Mean SANS_Attention_T0
5.00
4.00
3.00
2.00
1.00
0.00
Impaired Cluster
Preserved Cluster

1.19 9 month remission between group comparison

		Remission		
		Remitted	Unremitted	Total
Cluster	Preserved	33	65	98
	Impaired	41	68	109
Total		74	133	207

Chi-Square Tests

	Value	df	Asymptotic Significance (2-sided)	Exact Sig. (2-sided)	Exact Sig. (1-sided)
Pearson Chi-Square	.349[a]	1	.555		
Continuity Correction[b]	.198	1	.656		
Likelihood Ratio	.349	1	.554		
Fisher's Exact Test				.565	.328
Linear-by-Linear Association	.347	1	.556		
N of Valid Cases	207				
